# Comparative Outcomes of Distal Femoral Replacement Versus Operative Fixation for Periprosthetic Distal Femur Fractures: A Systematic Review and Meta-Analysis

**DOI:** 10.7759/cureus.111801

**Published:** 2026-06-30

**Authors:** Wazzan Aljuhani, Batool A Al Hussain, Osama A Moshantaf, Maha A AlDaher, Rashed M Alshammari, Abdullah Y Alheraiz, Lina F Almutairi, Salma A AlDaher, Zainab A Alsaffar, Noor A Abualsaud, Yaqeen M Almahozi, Omar Y Alhati, Mohammad K Albouri, Saied Y Sharif

**Affiliations:** 1 Orthopaedic Surgery, Ministry of National Guard Health Affairs (MNGHA), Riyadh, SAU; 2 Medical Research, King Abdullah International Medical Research Center, Riyadh, SAU; 3 College of Medicine, King Saud bin Abdulaziz University for Health Sciences, Riyadh, SAU; 4 College of Medicine, Vision College, Riyadh, SAU

**Keywords:** distal femoral replacement, intramedullary nail, open reduction internal fixation, periprosthetic distal femur fracture, plating

## Abstract

Periprosthetic distal femur fractures (PDFFs) are challenging injuries that commonly affect elderly patients following total knee arthroplasty (TKA). The commonly used surgical interventions are distal femoral replacement (DFR) and open reduction and internal fixation (ORIF), although the best approach remains debatable. This study aimed to evaluate the different ORIF subtypes and compare DFR versus ORIF for the management of PDFFs.

This study was conducted in strict accordance with the Preferred Reporting Items for Systematic Reviews and Meta-Analyses (PRISMA) guidelines and was registered with International Prospective Register of Systematic Reviews (PROSPERO). A comprehensive search was conducted across various databases up to October 2025. This study included only cohort studies comparing various surgical management approaches. Primary outcomes included functional scores (Knee Society Score (KSS)/Knee Society Function Score (KSFS)/Oxford Knee Score (OKS)), reoperation, and range of motion (ROM). Risk of bias and methodological quality were assessed using the Methodological Index for Non-Randomized Studies (MINORS) tool.

Fifteen retrospective comparative studies involving 1,417 patients were included. Pooled analysis demonstrated a statistically significant reduction in reoperation rates favoring DFR (OR = 0.64; 95% CI: 0.42-0.97). One-year mortality showed a non-significant trend favoring DFR (OR = 0.70; 95% CI: 0.45-1.09). Infection rates were slightly higher in the DFR group without statistical significance (OR = 1.39; 95% CI: 0.61-3.18). No significant differences were observed in perioperative and functional outcomes between the two interventions. DFR allowed earlier full weight-bearing and mobilization, whereas ORIF required protected weight-bearing protocols.

Compared with ORIF in PDFFs, DFR was associated with a reduced risk of reoperation and earlier postoperative mobilization. Mortality and functional outcomes remained comparable between the two interventions. Treatment selection should be individualized, with ORIF appropriate for reconstructible fractures with stable implants and DFR reserved for patients in whom fixation is unlikely to succeed or protected weight-bearing is unrealistic; all findings should be interpreted cautiously given the retrospective evidence base.

## Introduction and background

Periprosthetic distal femur fractures (PDFFs) are challenging injuries after total knee arthroplasty (TKA), commonly affecting elderly patients [1,2]. These fractures are particularly challenging to treat in older, frail individuals with osteoporosis, since these injuries are associated with high rates of major morbidity, impaired mobility, and significant mortality [1,3]. As a result, enhancing surgical treatment methods for PDFFs remains a clinically significant and emerging area of orthopedic research.

Traditional surgical approaches for managing PDFFs include open reduction and internal fixation (ORIF) with locking plates and retrograde or antegrade intramedullary nail (IMN) fixation. These represent commonly utilized surgical strategies for distal femoral fractures [1,4]. ORIF is considered successful for fracture healing; however, in complicated cases such as severe comminution, poor bone quality, and periprosthetic implant-related issues, it can lead to delayed healing. The elevated rates of non-union, fixation failure, and reoperation represent well-recognized limitations of ORIF in this patient population [1,5].

An alternative surgical approach for managing PDFFs is distal femoral replacement (DFR). Previous studies indicate that DFR is usually reserved for cases with significant bone loss, unstable or loose prosthetic components, or severely comminuted fractures that cannot be adequately reduced. DFR provides immediate mechanical stability, eliminates the requirement for fracture union, and allows early weight-bearing, which is useful in frail elderly patients [6,7]. In contrast, Wadhwa H et al. reported that DFR had greater concerns regarding surgical complexity, infection risk, and long-term implant survivability than ORIF [2].

Furthermore, recent comparative studies and systematic reviews have reported inconsistent findings regarding the outcomes of DFR and ORIF. Several studies indicate that ORIF and DFR have similar rates of complications and reoperations, despite variations in functional outcomes and postoperative complication profiles [1,8,9]. Nevertheless, the retrospective nature of the current evidence, as well as significant variation in patient selection, fracture classification, and outcome reporting, limits the ability to draw decisive conclusions [1]. In clinical practice, the decision between DFR and ORIF is rarely based on a single outcome; instead, it requires a multifactorial evaluation encompassing fracture reconstructibility, femoral component stability, residual bone stock, patient frailty, comorbidity burden, and the realistic potential for postoperative rehabilitation. It is within this complex clinical context that comparative outcome data must be interpreted and applied. This study aims to evaluate the clinical, functional, and perioperative outcomes of DFR versus ORIF for PDFFs, with additional assessment of various ORIF fixation subtypes, to better inform individualized surgical decision-making in this challenging patient population.

## Review

Methods and materials

Study Design and Registration

This study was conducted as a systematic review and meta-analysis in accordance with the Preferred Reporting Items for Systematic Reviews and Meta-Analyses (PRISMA) 2020 guidelines. The review was prospectively registered in the International Prospective Register of Systematic Reviews (PROSPERO) before the commencement of data extraction (Registration ID: CRD420251207262; registration date: 10 November 2025). The full protocol is available through PROSPERO and will be published upon completion of the review. No amendments to the pre-registered protocol were made following registration.

Eligibility Criteria

Studies were considered eligible based on a pre-specified Population-Intervention-Comparison-Outcome (PICO) framework, as detailed below.

Population: The review included studies enrolling adult patients aged ≥18 years with a confirmed diagnosis of PDFFs around total or unicompartmental knee arthroplasty. Studies that provided results separately for relevant operative treatment groups or provided adequate aggregate data for comparative extraction were considered eligible. Studies were excluded if they enrolled pediatric patients aged <18 years, exclusively addressed fractures not involving the distal femur, or included patients with associated pathological or tumor-related fractures.

Intervention: A variety of operative surgical treatments for PDFFs qualified as interventions. DFR, distal femoral arthroplasty (DFA), ORIF, locking compression plate (LCP) fixation, lateral locking plate (LLP) fixation, retrograde intramedullary nailing (RIMN), dual plating constructs, angular stable plate fixation, implant exchange procedures, and other standardized surgical fixation or reconstruction techniques were among the accepted surgical modalities. Studies that assessed conservative or non-operative treatment or isolated revision arthroplasty without a periprosthetic fracture procedure were excluded.

Comparison: The comparator of interest was alternative surgical interventions, including studies comparing various ORIF fixation methods and DFR/DFA with ORIF. Studies that lacked an active comparator group were excluded, as were single-arm studies with no comparator group or studies with irrelevant comparisons.

Outcomes: Primary outcomes included functional recovery, assessed by validated scoring systems, such as the Knee Society Score (KSS), Knee Society Functional Score (KSFS), and Oxford Knee Score (OKS), reoperation rate, and postoperative range of motion (ROM). Secondary outcomes included complication rates, such as one-year mortality, infection rate, non-union rate, and intraoperative blood loss, as well as implant survivorship and hospital length of stay.

Study design: Randomized controlled trials (RCTs) and prospective or retrospective comparative cohort studies with two or more comparative arms were included. Study designs such as case reports, case series, letters, editorials, commentaries, narrative reviews, conference abstracts without full-text data, and single-arm studies were excluded, as were studies published in languages other than English.

Information Sources and Search Strategy

**Figure 1:**
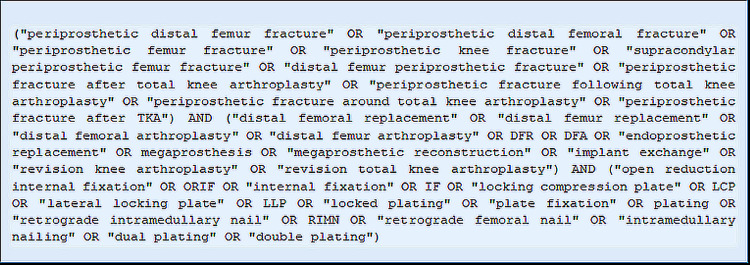
Boolean search string.

A comprehensive electronic literature search was conducted in PubMed, Google Scholar, and the Cochrane Central Register of Controlled Trials (CENTRAL) from database inception through the search date, with no publication date restrictions. The search strategy was constructed using a combination of MeSH terms and free-text keywords organized around three core conceptual domains: (1) PDFFs, (2) DFR, and (3) ORIF techniques, such as plate and IMN constructs. The full search strategy, including Boolean operators and database-specific adaptations, is provided in Figure 1 to ensure reproducibility.

Additional sources, such as the reference lists of all included studies through backward citation searching and the reference lists of pertinent systematic reviews and clinical practice guidelines identified during the search, were manually searched to reduce the risk of missing relevant research. When additional clarification of published data was needed, direct communication with content experts was considered. Grey literature, conference abstracts, and unpublished data were excluded. After the primary database search cut-off in October 2025, manual citation searching identified one additional reference, Brady T et al. [1], which was included because of its direct relevance to the primary comparison.

Study Selection

Initially, all records obtained from the electronic database searches were loaded into Rayyan, a web-based collaborative systematic review tool, and deduplicated. In two sequential phases, two reviewers independently reviewed the studies. The pre-established inclusion criteria were used to screen titles and abstracts in the initial phase. In the second phase, the same two reviewers retrieved and independently assessed the full texts of all potentially eligible records.

Disagreements at either phase were resolved through structured discussion between the two reviewers. If consensus could not be reached, a third independent reviewer was consulted for adjudication. Reasons for exclusion at the full-text stage were systematically recorded and are reported in the PRISMA flow diagram. Inter-reviewer agreement was monitored throughout the selection process.

Data Extraction

At least two reviewers independently extracted data using a standardized, pre-piloted Microsoft Excel extraction form. If required, a third reviewer was consulted, and the original publication was discussed to resolve any discrepancies. When relevant outcome data were not available in the published articles, the corresponding authors were approached to request the information. The following variables were extracted from each included study (Table 1).

**Table 1:** Data extraction summary. PROSPERO: International Prospective Register of Systematic Reviews; DFR: Distal femoral replacement; ORIF: Open reduction and internal fixation; IMN: Intramedullary nail/intramedullary nailing; RIMN: Retrograde intramedullary nail/retrograde intramedullary nailing; DFA: Distal femoral arthroplasty; KSS: Knee Society Score; KSFS: Knee Society Functional Score; OKS: Oxford Knee Score; ROM: Range of motion; PJI: Periprosthetic joint infection.

Domain	Variables Extracted
Study characteristics	First author, year of publication, journal, country of origin, study design, level of evidence, PROSPERO/trial registration number, funding source, and follow-up duration.
Participant demographics	Total sample size, group allocation (DFR vs. ORIF or ORIF vs. ORIF), mean age ± SD, age range, sex distribution, BMI, comorbidities (e.g., diabetes and osteoporosis), and mechanism of injury.
Clinical and fracture characteristics	Surgical indication (periprosthetic distal femur fracture), fracture classification system, condition of the femoral component (stable vs. loose), fracture pattern, and interval between injury and surgery.
Intervention details	ORIF: fixation method (locking plate, IMN, dual plating, or combined constructs), surgical approach, and technical variations reported across studies.
Comparison details	ORIF: surgical technique, technical variations reported across studies, and fixation method (locking plate, RIMN, dual plating, or combined constructs). DFR/DFA: prosthesis type (megaprosthesis or revision arthroplasty system) and any relevant surgical details reported.
Functional outcomes	Knee Society Score (KSS), Knee Society Functional Score (KSFS), Oxford Knee Score (OKS), and range of motion (ROM).
Complications and adverse events	Reoperation rate, revision surgery, implant failure, and aseptic loosening. Surgical site infection, periprosthetic joint infection (PJI), and deep infection. One-year mortality, 30-day mortality, and overall mortality rates. Non-union rate in the ORIF group and fracture-healing complications.
Perioperative outcomes	Intraoperative blood loss (mL) and length of hospital stay.
Postoperative recovery outcomes	Return to daily activities, weight-bearing status, time to full weight-bearing, and rehabilitation protocols.

To facilitate both short- and long-term comparisons across studies, data were retrieved for all available follow-up intervals.

Risk of Bias Assessment

Two qualified reviewers independently assessed the methodological quality of each included study; differences were resolved by discussion or by a third reviewer. MINORS scores ranged from 12/24 to 20/24, indicating overall moderate methodological quality across the included evidence base.

Non-randomized comparative studies (MINORS): All studies included in this review were retrospective and non-randomized, including retrospective comparative case series and retrospective cohort studies. The MINORS scores, which ranged from 12/24 to 20/24, indicated that the methodological quality of the evidence base was generally consistent. Ross LA et al. [10] obtained the highest score (20/24), whereas Tandon T et al. [11], Leino OK et al. [12], and Ruder JA et al. [13] received the lowest scores (12/24). The methodological quality of the included studies was considered moderate overall. The lower scores primarily reflected the research design and reporting quality. Despite these limitations, in the absence of randomized controlled trials, the included studies provide the strongest comparative evidence on this subject (Table 2).

**Table 2:** Summary of risk-of-bias assessments for all included studies. MINORS: Methodological Index for Non-Randomized Studies.

#	Study (Author, Year)	Study Design	MINORS Overall Score
1	Ross LA et al. (2021) [10]	Retrospective cohort study	20/24
2	Tandon T et al. (2020) [11]	Retrospective comparative cohort study	12/24
3	Leino OK et al. (2015) [12]	Retrospective cohort study	12/24
4	Ruder JA et al. (2017) [13]	Retrospective cohort study	12/24
5	Aldrian S et al. (2013) [14]	Retrospective comparative case series	16/24
6	Abboud J et al. (2024) [15]	Retrospective multicenter comparative cohort study	13/24
7	Battut T et al. (2022) [16]	Retrospective comparative case series	12/24
8	De Marco D et al. (2022) [17]	Retrospective cohort study	12/24
9	Hoellwarth JS et al. (2018) [18]	Retrospective cohort study	16/24
10	Fu P et al. (2022) [19]	Retrospective comparative cohort study	14/24
11	Darrith B et al. (2020) [20]	Retrospective cohort study	17/24
12	Lizcano JD et al. (2025) [21]	Retrospective cohort study	13/24
13	Kriechling P et al. (2024) [22]	Retrospective cohort study	16/24
14	Virkus W et al. (2022) [23]	Retrospective comparative case series	18/24
15	Gausden EB et al. (2021) [24]	Retrospective cohort study	13/24
Level of evidence was classified according to the Oxford Centre for Evidence-Based Medicine (OCEBM). MINORS was scored out of 24 points, with 12 items scored up to 2 points each. Higher scores indicate better methodological quality.			

Reporting Bias

The potential for reporting bias was evaluated at two levels. At the outcome level, selective reporting was evaluated by comparing the results reported in the Results section with those declared in the Methods section of each included study. The evaluation of selective reporting was based solely on the consistency of reported endpoints across publications, as all included studies were retrospective observational studies and had no prospectively documented methods. At the review level, publication bias for meta-analyzed outcomes was examined using funnel plot asymmetry, with a minimum of ten studies contributing data to the pooled estimate, and statistical testing was performed using the Egger regression intercept test.

Certainty of Evidence

The overall certainty of the evidence for each primary and secondary outcome was evaluated using the Grading of Recommendations, Assessment, Development, and Evaluation (GRADE) framework. The evidence was categorized as high, moderate, low, or very low certainty and assessed across five domains: risk of bias, inconsistency, indirectness, imprecision, and publication bias. Evidence from the included observational studies (Level III-IV) began at low certainty and could be upgraded if specific conditions were met. Summary of Findings (SoF) tables were generated for the primary outcomes.

Data Synthesis and Statistical Analysis

Quantitative synthesis: When two or more studies reported data for the same outcome in a sufficiently homogeneous manner, both clinically and methodologically, a meta-analysis was performed using a random-effects model (DerSimonian and Laird method) to account for between-study heterogeneity. For dichotomous outcomes, such as reoperation, mortality, infection, and non-union, risk ratios (RRs) or ORs with 95% CIs were calculated. ORs were selected as the primary effect measure given the retrospective nature of all included studies and the low event rates observed across most outcomes. A continuity correction of 0.5 was applied to studies reporting zero occurrences in one or more arms, enabling these studies to be included in the pooled analysis. For continuous outcomes, such as intraoperative blood loss and length of hospital stay, the mean difference (MD) or standardized mean difference (SMD) was used as appropriate. All analyses were conducted using Review Manager (RevMan) version 5.4 (Cochrane Collaboration).

Heterogeneity: The Cochran Q test (chi-squared) was used in conjunction with the I² statistic to quantify statistical heterogeneity. Low, moderate, and high heterogeneity were indicated by I² values of less than 25%, 25%-50%, and greater than 50%, respectively. Potential sources of heterogeneity were investigated using pre-specified subgroup analyses and sensitivity analyses where significant heterogeneity (I² >50%) was found. For outcomes demonstrating very high heterogeneity (I² >75%), pooled estimates are presented descriptively and should not be used as precise clinical benchmarks; narrative synthesis was prioritized for such outcomes to avoid misleading conclusions.

Subgroup analyses: Pre-planned subgroup analyses were conducted to evaluate the potential moderating effects of (1) patient age group (adults aged 18 years and older), (2) surgical technique (DFR vs. ORIF) or types of ORIF (LP vs. retrograde IMN vs. dual plating), (3) fracture classification system (Su vs. Rorabeck vs. other classifications), and (4) study design (retrospective cohort studies vs. retrospective comparative case series). Given the expected small number of studies in each class, these subgroup analyses were considered exploratory.

Sensitivity analyses: Sensitivity analyses were performed by sequentially excluding studies rated as scoring below 14 on the MINORS scale to evaluate the robustness of pooled estimates to study quality. Additional sensitivity analyses were conducted by excluding the oldest study [14]. The robustness of the results was confirmed by the pooled results, which didn’t vary in direction or magnitude.

Narrative synthesis: A structured narrative synthesis was provided for results for which meta-analysis was not possible due to inadequate data, significant clinical heterogeneity, or the use of non-comparable assessment tools. Descriptive summaries of the results were provided, and tables were used to facilitate cross-study comparisons. When appropriate, recurring patterns of results were observed, and the direction and strength of effects were qualitatively described.

Results

Study Selection and Characteristics

A total of 15 comparative studies encompassing 1,417 patients were identified and included in the final analysis following the systematic database search and screening process. The included studies were published between 2013 and 2025, spanning 12 years of comparative surgical literature on PDFFs. The studies originated from seven countries, including the United States (n = 7), the United Kingdom (n = 3), France (n = 2), Italy (n = 1), China (n = 1), Finland (n = 1), and Austria (n = 1), reflecting broad international representation.

All 15 included studies were retrospective in design, comprising retrospective cohort studies (n = 12) and retrospective comparative case series (n = 3). The level of evidence was Level III in 12 studies and Level IV in three studies [14,15,16], as classified according to the Oxford Centre for Evidence-Based Medicine criteria. No prospective, randomized, or pseudo-randomized studies were identified. The exclusively retrospective nature of the available evidence must be considered a fundamental interpretive constraint throughout this review, rendering all pooled comparisons vulnerable to selection bias, confounding by indication, and unmeasured patient-level variables that prospective or randomized designs would otherwise control. Study sample sizes ranged from 13 patients in De Marco D et al. [17] to 434 patients in Abboud J et al. [15]. Follow-up duration varied widely across the studies, ranging from six weeks in Aldrian S et al. [14] to six years in Tandon T et al. [11].

**Figure 2:**
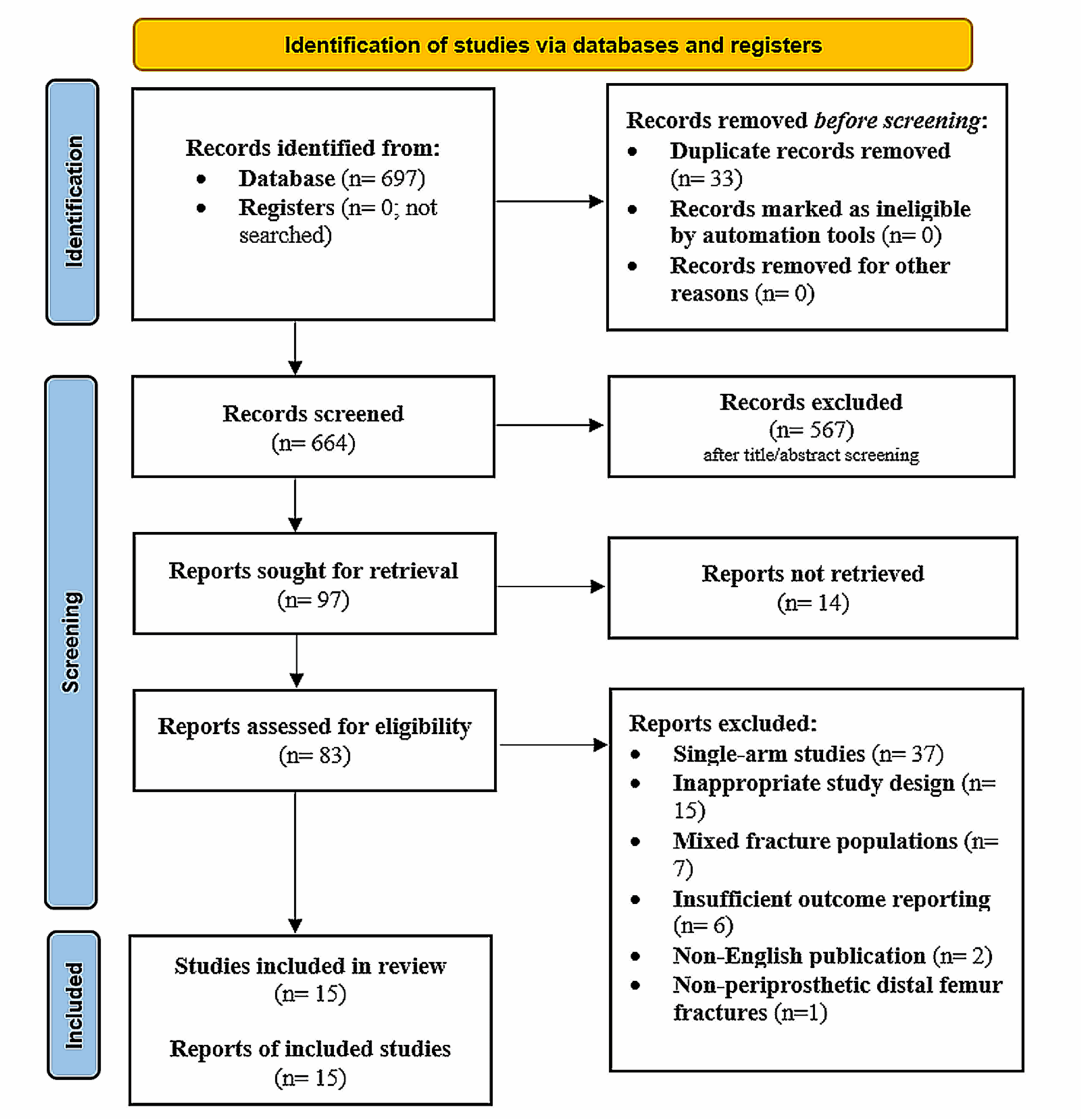
PRISMA flowchart. PRISMA: Preferred Reporting Items for Systematic Reviews and Meta-Analyses.

Of the 15 included studies, 11 provided direct comparisons between DFR and ORIF [10-13,16-22]. Four studies [14,15,23,24] were included for subgroup analysis of ORIF fixation types, including locked plate versus RIMN versus dual plating, and did not include a DFR comparator group. Consequently, findings from these studies should be interpreted in the context of variation in outcomes across fixation constructs, rather than as direct comparative evidence between DFR and ORIF (Figure 2).

Methodological quality assessment: Methodological quality was assessed using the Methodological Index for Non-Randomized Studies (MINORS) tool, with a maximum attainable score of 24 points. Across the 15 included studies, MINORS scores ranged from 12 to 20 out of 24 points. The highest score was recorded for Ross LA et al. (20/24) [10], while the lowest scores (12/24) were reported in studies [11-13,16,17]. The mean MINORS score across all studies was approximately 14.4/24, and the median was 13/24, indicating fair-to-moderate overall methodological quality. Common sources of methodological weakness across studies included the absence of prospective data collection (item 3), lack of unbiased endpoint assessment (item 5), loss to follow-up exceeding 5% (item 7), and absence of prospective sample size calculations (item 8). Notably, no study reported a prospective sample size calculation, reflecting the universally retrospective nature of the included evidence base. These recurring limitations reduce confidence in the precision of effect estimates, particularly for outcomes with low event rates such as infection, mortality, and revision surgery, where small absolute numbers amplify the risk of imprecise conclusions (Table 3).

**Table 3:** Quality assessment of included studies. MINORS: Methodological Index for Non-Randomized Studies (Slim K et al. (2003)). Each item was scored as 0 (not reported), 1 (reported but inadequate), or 2 (reported and adequate). The maximum score was 24 points.

#	Study	Study Design			Data Collection					Comparative Studies				Total / 24
		1	2	3	4	5	6	7	8	9	10	11	12	
1	De Marco D et al. (2022) [17]	2	0	0	2	2	2	0	0	0	0	2	2	12/24
2	Ross LA et al. (2021) [10]	2	2	1	2	2	2	2	0	2	2	1	2	20/24
3	Hoellwarth JS et al. (2018) [18]	2	2	0	2	0	2	0	0	2	2	2	2	16/24
4	Tandon T et al. (2020) [11]	2	1	1	2	0	2	0	0	1	2	1	0	12/24
5	Fu P et al. (2022) [19]	2	2	0	2	0	1	1	0	1	2	1	2	14/24
6	Leino OK et al. (2015) [12]	2	1	0	2	0	2	0	0	1	2	1	1	12/24
7	Darrith B et al. (2020) [20]	2	2	0	2	1	2	2	0	2	1	1	2	17/24
8	Lizcano JD et al. (2025) [21]	2	1	0	2	1	1	0	0	1	2	1	2	13/24
9	Ruder JA et al. (2017) [13]	2	1	0	2	1	1	0	0	1	2	1	1	12/24
10	Kriechling P et al. (2024) [22]	2	2	1	2	1	1	1	0	1	2	1	2	16/24
11	Abboud J et al. (2024) [15]	2	1	0	2	0	2	1	0	1	2	1	1	13/24
12	Battut T et al. (2022) [16]	2	1	0	2	0	1	1	0	1	2	1	1	12/24
13	Virkus W et al. (2022) [23]	2	1	0	2	1	2	0	0	2	2	2	2	18/24
14	Gausden EB et al. (2021) [24]	2	1	0	2	1	1	1	0	1	2	1	1	13/24
15	Aldrian S et al. (2013) [14]	2	1	1	2	0	2	2	0	1	2	1	2	16/24

Baseline Patient Characteristics

The baseline patient characteristics are summarized in Table 4. The mean age of patients across studies ranged from 69.9 to 83 years, with most cohorts having a mean age between 75 and 81 years. Female patients predominated in all studies, comprising between 69% and 89% of the cohorts. BMI was reported in five studies and ranged from 24.8 to 34 kg/m².

**Table 4:** Baseline patient characteristics. DFR: Distal femoral replacement; ORIF: Open reduction and internal fixation; DFA: Distal femoral arthroplasty; LCP: Locking compression plate; LLP: Lateral locking plate; RIMN: Retrograde intramedullary nail; IMN: Intramedullary nail; CCI: Charlson Comorbidity Index; CHF: Congestive heart failure; CKD: Chronic kidney disease; COPD: Chronic obstructive pulmonary disease; DM: Diabetes mellitus; RA: Rheumatoid arthritis; Su: Su classification; OTA: Orthopaedic Trauma Association; ASA: American Society of Anesthesiologists; SOFCOT: Société Française de Chirurgie Orthopédique et Traumatologique; IE: Implant exchange; IF: Internal fixation; ASP: Angular stable plate; RIN: Rigid interlocking nail; NR: Not reported. Note: Abboud J et al. (2024) [15], Virkus W et al. (2022) [23], Gausden EB et al. (2021) [24], and Aldrian S et al. (2013) [14] did not include a DFR group and compared different fixation methods only. Kriechling P et al. (2024) [22] compared DFA versus double plating (DP) versus single plating (SP). Percentages for sex were calculated from the reported sample size where the total sample size was provided.

#	Study (Author, Year)	Mean Age	Sex (M/F)	BMI (kg/m²)	Osteoporosis	Diabetes	Smoker	Fracture Classification	Comorbidities/Notes
1	De Marco D et al. (2022) [17]	ORIF: 73.4 (SD NR); DFR: 74.75 (SD NR)	M: 4 (31%); F: 9 (69%)	27.4 ± 4.2	NR	DM: 3	NR	Su	CKD; DM; COPD; dementia; hemiplegia; CHF
2	Ross LA et al. (2021) [10]	ORIF: 81.3 ± 10.5; DFR: 78.8 ± 8.3	M: 12 (20%); F: 48 (80%)	ORIF: 26.7 ± 5.5; DFR: 26.7 ± 6.6	ORIF: 30%; DFR: 52%	NR	NR	Su	NR
3	Hoellwarth JS et al. (2018) [18]	LLP: 80.0 ± 9.9; DFR: 80.1 ± 7.8	NR	LLP: 32.6 ± 9.3; DFR: 30.6 ± 7.8	NR	LLP: 11 with end-organ damage; DFR: 0	LLP: 5; DFR: 3	OTA-33/Su	LLP: diabetes with end-organ damage
4	Tandon T et al. (2020) [11]	DFR: 78 (SD NR); ORIF: 74 (SD NR)	M: 12 (20%); F: 49 (80%)	NR	NR	NR	NR	Kim	Elderly osteoporotic females
5	Fu P et al. (2022) [19]	LCP: 69.9 ± 11.7; DFR: 72.7 ± 7.6	M: 2 (11%); F: 16 (89%)	NR	NR	NR	NR	NR	ASA classification used
6	Leino OK et al. (2015) [12]	78.9 ± 8.2	M: 10 (15%); F: 58 (85%)	NR	42.70%	10.30%	NR	Rorabeck	CHF: 33.8%; Alzheimer’s disease: 19.1%; Parkinson’s disease: 4.4%; stroke: 8.8%; RA: 17.7%
7	Darrith B et al. (2020) [20]	DFR: 75.8 ± 8.4; ORIF: 71.8 ± 10.6	DFR: M: 14%; F: 86%; ORIF: M: 12%; F: 88%	NR	NR	NR	NR	Rorabeck type II	Comorbidity burden/CCI: DFR: 5.2; ORIF: 3.8
8	Lizcano JD et al. (2025) [21]	DFR: 78 ± 10.3; ORIF: 70 ± 11.4	DFR: M: 81.5%; ORIF: M: 80.8%	32.3 ± 7.9	NR	NR	ORIF: 1%	Su	CCI: DFR: 5.4; ORIF: 3.8
9	Ruder JA et al. (2017) [13]	DFR: 83; ORIF: 78	M: 12 (21%); F: 46 (79%)	NR	NR	NR	NR	NR	NR
10	Kriechling P et al. (2024) [22]	Median: 82 (IQR, 75–88); DFA: 83; DP: 78; SP: 83	M: 15 (14%); F: 96 (86%)	NR	DFA: 67%; DP: 33%; SP: 47%	DFA: 15%; DP: 27%; SP: 9.4%	NR	Su/Fakler	Osteoporosis: 53; DM: 14; steroid use: 12
11	Abboud J et al. (2024) [15]	75 ± 2	M: 208 (48%); F: 226 (52%)	LP: 26 ± 2; IMN: 24.8 ± 1	NR	NR	NR	SOFCOT	NR
12	Battut T et al. (2022) [16]	IE: 80 (SD NR); IF: 84.5 (SD NR)	NR	NR	NR	NR	NR	AO/SOFCOT	NR
13	Virkus W et al. (2022) [23]	RIMN: 73.3 ± 9.9; ORIF: 75.9 ± 11.4	RIMN: M: 3 (8%); ORIF: M: 1 (8%)	NR	NR	NR	NR	Su	NR
14	Gausden EB et al. (2021) [24]	Plating: 76; RIMN: 75	M: 25 (26%); F: 72 (74%)	Plating: 29; RIMN: 34 ± 2	NR	NR	NR	Su	NR
15	Aldrian S et al. (2013) [14]	75.6 (SD NR)	M: 24 (28%); F: 62 (72%)	NR	NR	NR	NR	Su	NR

Osteoporosis was variably reported across studies. Leino OK et al. [12] reported osteoporosis in 42.7% of the overall cohort. Kriechling P et al. [22] reported osteoporosis in 67% of the distal femoral arthroplasty group, 33% of the double-plating group, and 47% of the single-plating group. The prevalence of diabetes mellitus ranged from 9.4% to 27% across studies. Smoking status was infrequently reported.

Notable imbalances in comorbidity burden were observed between treatment groups. Lizcano JD et al. [21] reported a mean Charlson Comorbidity Index (CCI) of 5.4 in the DFR group versus 3.8 in the ORIF group. Similarly, Darrith B et al. [20] noted a higher comorbidity burden in DFR patients, with a mean CCI of 5.2 compared with 3.8 in ORIF patients. Leino OK et al. [12] reported substantial baseline comorbidities in the overall cohort, including congestive heart failure (33.8%), Alzheimer's disease (19.1%), rheumatoid arthritis (17.7%), and diabetes mellitus (10.3%). Hoellwarth JS et al. [18] reported diabetes with end-organ damage in 11 patients within the locked plating group.

Fracture classification systems varied across studies. The Su classification was used by studies [11,14,17,22,23,24]. The Rorabeck classification was used by studies [12,20]. The Kim classification was used by Tandon T et al. [11]. Abboud J et al. [15] used the SOFCOT classification. The differences in baseline characteristics observed between the treatment groups have important clinical implications, as patients selected for DFR had consistently more challenging fracture patterns, worse bone quality, and a higher burden of comorbid disease than those managed with ORIF. In the absence of propensity matching or randomization, these imbalances represent a meaningful risk of confounding.

Meta-Analysis of Primary and Secondary Outcomes

**Figure 3:**
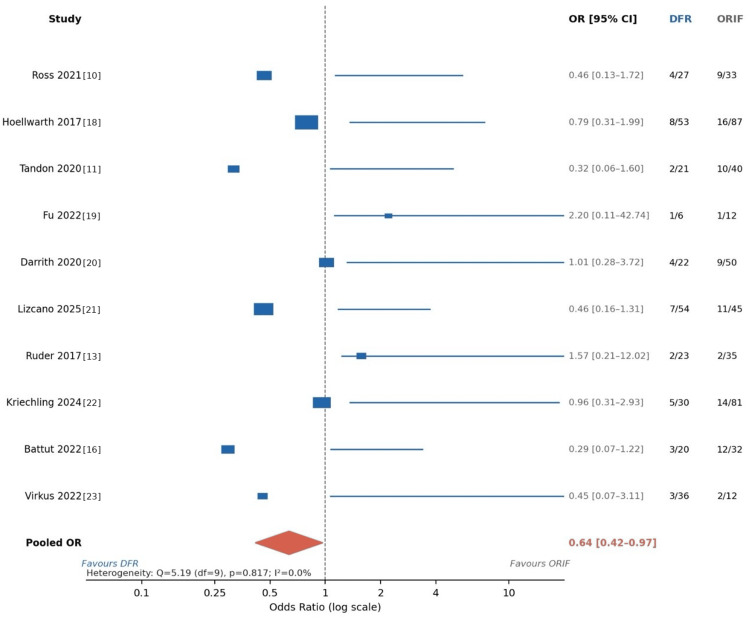
Forest plot of the odds ratio for reoperation rate comparing distal femoral replacement (DFR) with open reduction and internal fixation (ORIF).

Reoperation rate: Ten studies [10,11,13,16,18,19,20,21,22,23] contributed to the meta-analysis of reoperation rate. The pooled OR for reoperation in DFR versus ORIF was 0.64 (95% CI: 0.42-0.97), indicating a statistically significant reduction in the odds of reoperation among patients treated with DFR compared with ORIF (Figure 3). This result represents a clinically meaningful finding favoring DFR for the primary outcome measure of this review; however, given the retrospective design of all contributing studies, the observed reduction in reoperation rates should be interpreted as an association rather than definitive evidence of DFR’s superiority over ORIF. Across the ten contributing studies, absolute reoperation rates ranged from 7%-10% in DFR cohorts and 18%-24% in ORIF cohorts, although rates were not directly poolable due to differences in outcome definitions. Heterogeneity was negligible (Q = 5.19, df = 9, p = 0.817; I² = 0.0%), indicating high consistency of effect estimates across studies.

Individual study estimates ranged from an OR of 0.29 [16] to 2.20 [19]. Fu P et al. [19] was a notable outlier with an OR of 2.20 (95% CI: 0.11-42.74), favoring ORIF. However, this estimate was derived from a small sample (6 DFR vs. 12 ORIF patients) and carried very wide CIs, limiting its interpretive weight. The majority of individual study estimates trended in the direction favoring DFR, including Tandon T et al. [11] (OR 0.32; 95% CI: 0.06-1.60), Ross L et al. [10] (OR 0.46; 95% CI: 0.13-1.72), Lizcano JD et al. [21] (OR 0.46; 95% CI: 0.16-1.31), and Battut T et al. (OR 0.29; 95% CI: 0.07-1.22) [16].

**Figure 4:**
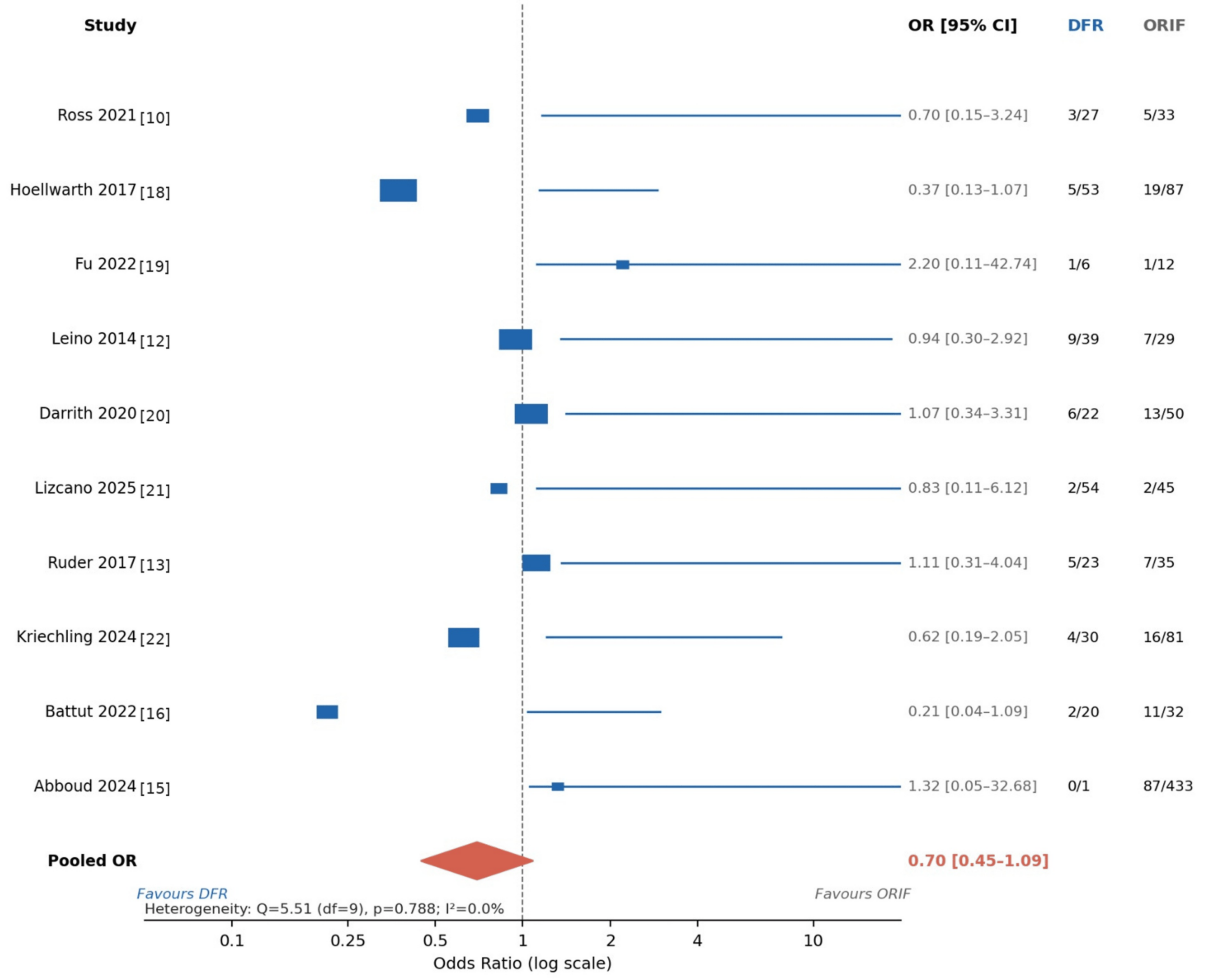
Forest plot of the odds ratio for one-year mortality comparing distal femoral replacement (DFR) with open reduction and internal fixation (ORIF).

One-year mortality: Ten studies [10,12,13,15,16,18,19,20,21,22] contributed to the pooled analysis of one-year mortality. The pooled OR for one-year mortality in DFR versus ORIF was 0.70 (95% CI: 0.45-1.09), indicating a trend toward lower mortality in DFR patients; however, this result did not reach statistical significance, as the CI crossed the null value of one (Figure 4). Where applicable, a continuity correction of 0.5 was applied to studies reporting zero events in one or more arms to enable their inclusion in the pooled analysis. Heterogeneity was low (Q = 5.51, df = 9, p = 0.788; I² = 0.0%).

Individual study estimates demonstrated variability in direction. Battut T et al. [16] reported the largest observed mortality benefit for DFR (OR 0.21; 95% CI: 0.04-1.09), while Fu P et al. [19] (OR 2.20; 95% CI: 0.11-42.74) and Abboud J et al. [15] (OR 1.32; 95% CI: 0.05-32.68) showed imprecise point estimates favoring ORIF, each with very wide CIs consistent with small event numbers. The lack of statistical significance for this outcome is acknowledged as potentially reflecting insufficient power, given the relatively low event rates across the included studies. Most importantly, mortality in this patient cohort is more a function of baseline frailty, pre-existing comorbidity burden, pre-injury functional status, and postoperative medical complications than surgical implant selection. The currently available retrospective data do not indicate a causal association between implant choice and mortality, and these results should be interpreted with caution.

**Figure 5:**
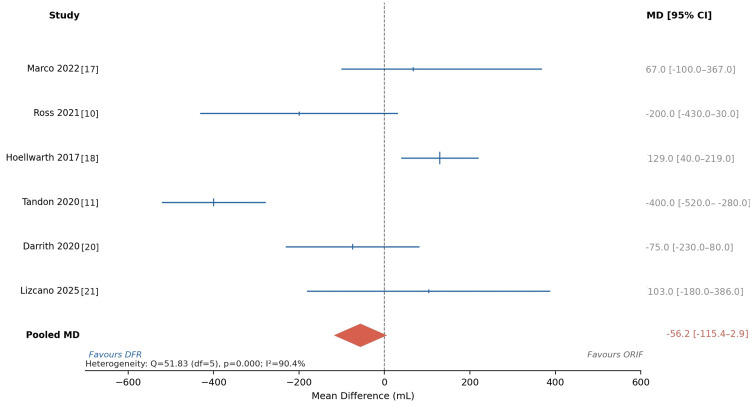
Forest plot of the mean difference in intraoperative blood loss (mL) comparing distal femoral replacement (DFR) with open reduction and internal fixation (ORIF). Negative values favour DFR.

Intraoperative blood loss: Six studies [10,11,17,18,20,21] reported intraoperative blood loss and were included in the pooled analysis. The pooled MD for intraoperative blood loss was -56.2 mL (95% CI: -115.4 to 2.9 mL), indicating a trend toward reduced blood loss with DFR compared with ORIF (Figure 5). This result did not achieve statistical significance, as the CI marginally crossed zero. Substantial heterogeneity was identified (Q = 51.83, df = 5, p < 0.001; I² = 90.4%), indicating marked inconsistency in blood loss data across studies.

The direction of effect was inconsistent at the level of individual studies. Tandon T et al. [11] reported the largest reduction in blood loss favoring DFR (MD -400 mL; 95% CI: -520 to -280 mL), whereas Hoellwarth JS et al. [18] reported significantly greater blood loss in DFR patients (MD +129 mL; 95% CI: 40 to 219 mL), consistent with the more extensive surgical dissection required for prosthetic replacement. The high heterogeneity suggests that blood loss outcomes are highly context-dependent, varying with surgical approach, implant type, patient bone quality, and transfusion thresholds across institutions. Multiple context-dependent factors, including surgical approach, case complexity, implant choice, surgeon experience, and institutional transfusion protocols, inherently influence intraoperative blood loss. Accordingly, this outcome should not serve as a primary determinant of treatment selection between DFR and ORIF, and the pooled estimate reported here should be interpreted with considerable caution.

**Figure 6:**
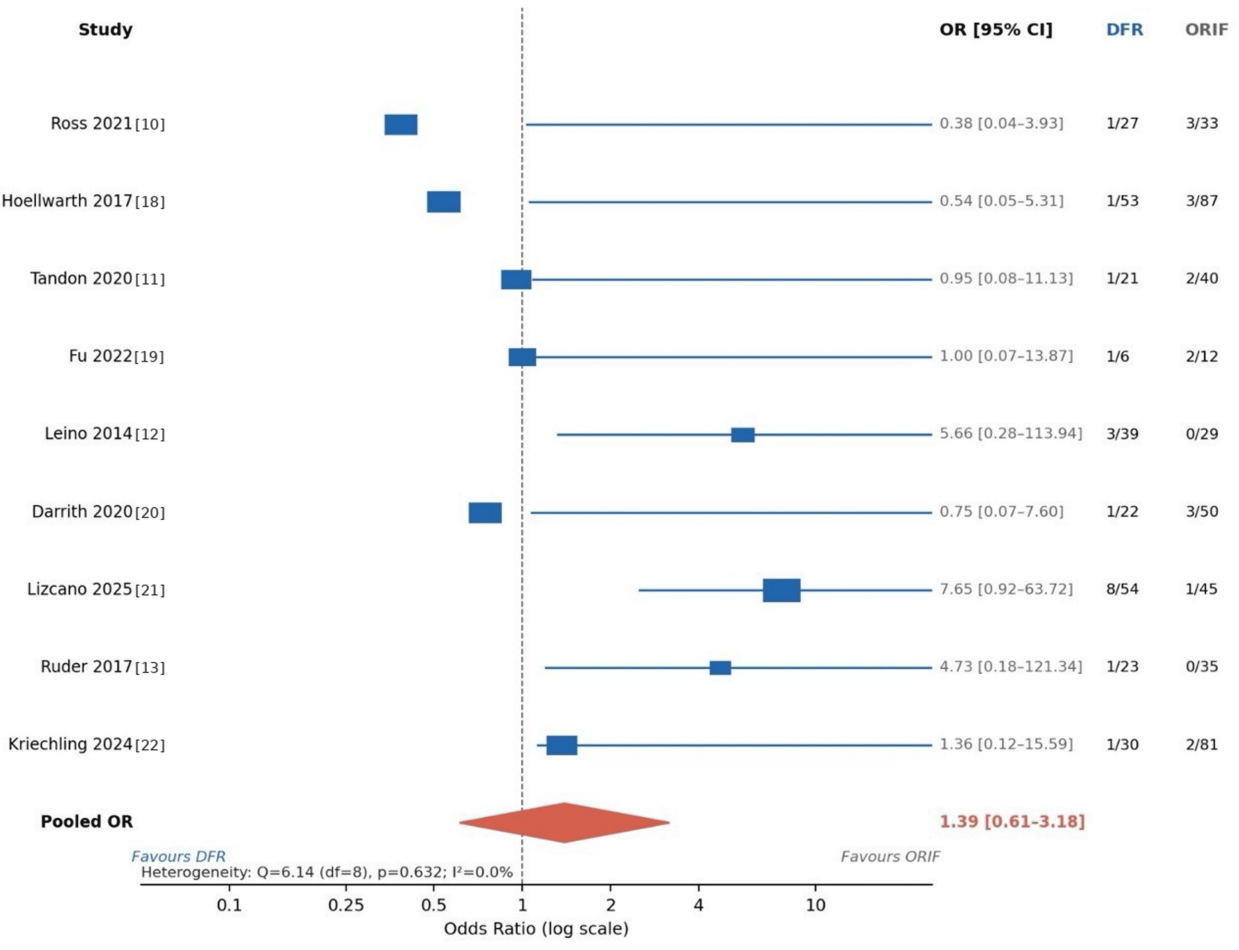
Forest plot of the odds ratio for postoperative infection rate comparing distal femoral replacement (DFR) with open reduction and internal fixation (ORIF).

Infection rate: Nine studies [10-13,18-22] were included in the meta-analysis of infection rate. The pooled OR for infection in DFR versus ORIF was 1.39 (95% CI: 0.61-3.18), indicating a non-significant trend toward higher infection risk in DFR patients, with the CI crossing one (Figure 6). A continuity correction of 0.5 was applied to studies reporting zero events in one or more arms to enable their inclusion in the pooled analysis. Heterogeneity was negligible (Q = 6.14, df = 8, p = 0.632; I² = 0.0%).

Lizcano JD et al. [21] (OR 7.65; 95% CI: 0.92-63.72) and Leino OK et al. [12] (OR 5.66; 95% CI: 0.28-113.94) reported the largest individual effect sizes, both favoring ORIF for infection risk, although neither reached statistical significance individually, likely due to small event numbers and correspondingly wide CIs. Conversely, Ross LA et al. [10] (OR 0.38; 95% CI: 0.04-3.93) and Hoellwarth JS et al. [18] (OR 0.54; 95% CI: 0.05-5.31) reported point estimates favoring DFR for infection. The absence of a statistically significant pooled effect precludes definitive conclusions regarding differential infection risk between the two interventions. Rather, this outcome is limited by the intrinsic constraints of this comparison, namely the small absolute number of infection events across contributing studies and the considerable variability in infection definitions and diagnostic criteria, both of which greatly restrict the statistical power and interpretive value of this outcome.

**Figure 7:**
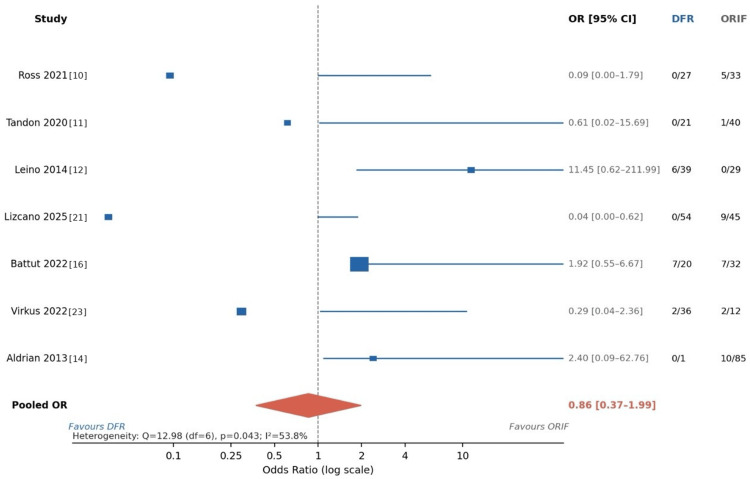
Forest plot of the odds ratio for non-union rate comparing distal femoral replacement (DFR) with open reduction and internal fixation (ORIF).

Non-union rate: Seven studies [10,11,12,14,16,21,23] contributed data on the non-union rate. This analysis is considered exploratory and methodologically limited: non-union is biologically inapplicable to DFR, given the absence of a fracture-healing requirement following prosthetic reconstruction, and studies that code DFR patients as having zero non-unions are comparing a structural impossibility with a biological event. The pooled OR for non-union in DFR versus ORIF was 0.86 (95% CI: 0.37-1.99), indicating no statistically significant difference between DFR and ORIF for this outcome (Figure 7); however, this result should not be interpreted as evidence of equivalent non-union risk. Moderate heterogeneity was present (Q = 12.98, df = 6, p = 0.043; I² = 53.8%).

The direction of individual study estimates was inconsistent. Lizcano JD et al. [21] reported a markedly lower non-union risk with DFR (OR 0.04; 95% CI: 0.00-0.62), as did Ross LA et al. [10] (OR 0.09; 95% CI: 0.00-1.79), reflecting that non-union is biologically not possible in DFR given the absence of a fracture-healing requirement following prosthetic replacement. Conversely, Leino OK et al. [12] reported an OR of 11.45 (95% CI: 0.62-211.99) favoring ORIF, although this estimate was imprecise. The distinct mechanisms underlying union and implant failure in each intervention group contribute to the observed heterogeneity. Notably, non-union and implant-related failure represent fundamentally different but functionally equivalent adverse outcomes within each treatment pathway; therefore, comparing non-union rates in ORIF against aseptic loosening, implant failure, and revision surgery in DFR would constitute a more clinically meaningful and biologically appropriate comparison.

**Figure 8:**
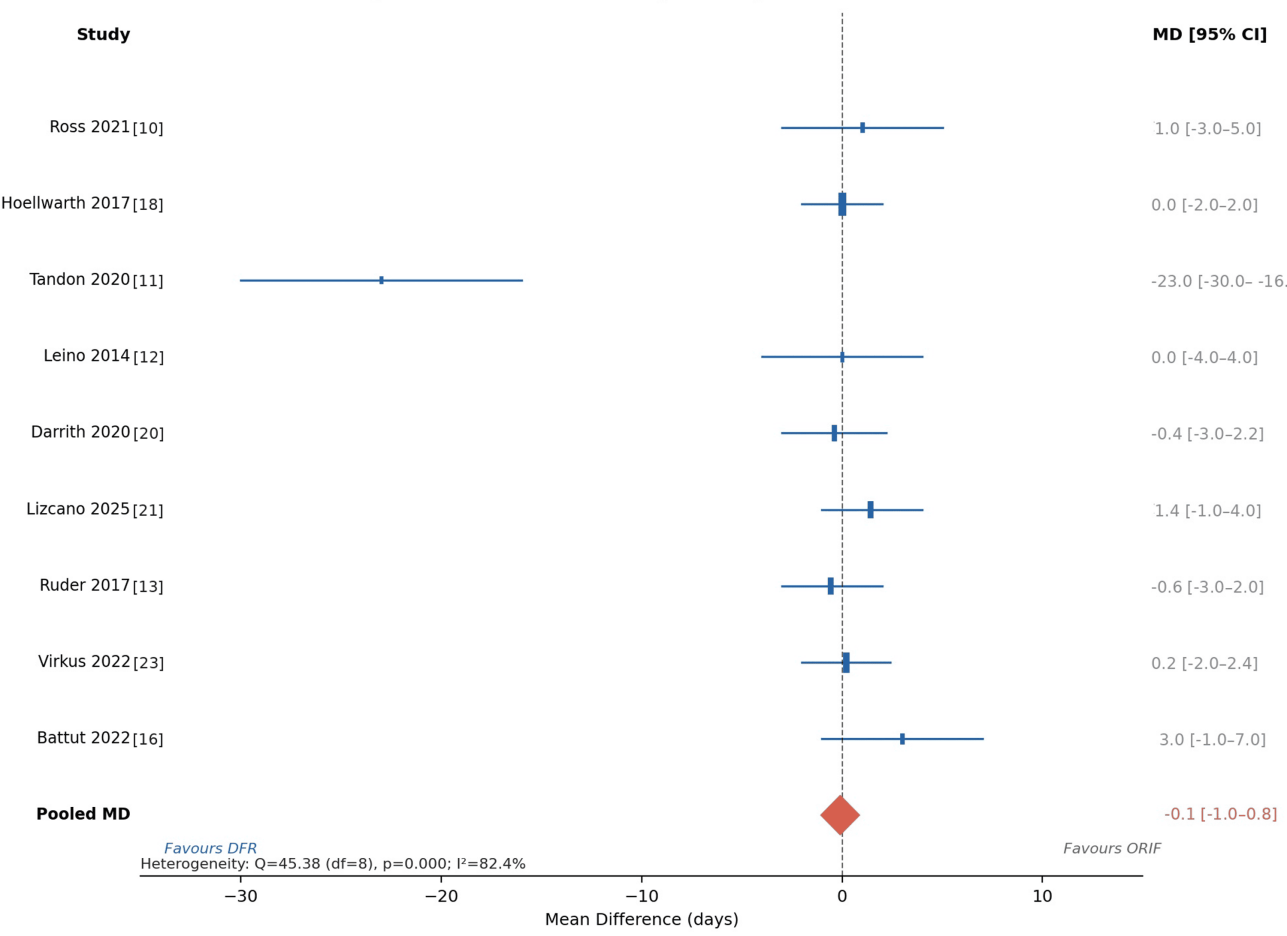
Forest plot of the mean difference in hospital stay (days) comparing distal femoral replacement (DFR) with open reduction and internal fixation (ORIF). Negative values favour DFR. DFR: Distal femoral replacement.

Hospital stay: Nine studies [10-13,16,18,20,21,23] reported length of hospital stay and were included in the meta-analysis. The pooled MD was -0.1 days (95% CI: -1.0 to 0.8 days), indicating no statistically significant difference in hospital length of stay between DFR and ORIF (Figure 8). Very high heterogeneity was identified (Q = 45.38, df = 8, p < 0.001; I² = 82.4%), suggesting that institutional protocols, patient case mix, and healthcare system factors exert substantial influence on this outcome.

Tandon T et al. [11] was a notable outlier, reporting a markedly shorter hospital stay for DFR compared with ORIF (MD -23.0 days; 95% CI: -30.0 to -16.0 days). Excluding this study, all remaining studies reported hospital stay differences of less than three days in either direction, with most CIs crossing zero. The extreme result reported by Tandon T et al. [11] may reflect differences in postoperative rehabilitation protocols, discharge criteria, or healthcare funding models specific to that institution. Length of hospital stay is primarily system-dependent. It cannot be meaningfully generalized across healthcare contexts, as it is determined by local discharge pathways, rehabilitation availability, and social care infrastructure rather than by surgical intervention alone.

Subgroup and Descriptive Analyses

Analysis by fixation type: The internal fixation methods used in the ORIF groups were categorized into three subgroups: locking plate fixation, including LCP, LLP, and angular stable plates; RIMN; and dual plating. Locking plate fixation was the most commonly reported method, used in 13 of 16 studies. RIMN was used in 12 studies, either as the primary fixation method or as a comparator. Dual plating was reported in six studies, most notably by Kriechling P et al. [22], who directly compared double plating versus single plating versus distal femoral arthroplasty.

Blood loss data varied by fixation type. Hoellwarth JS et al. [18] reported significantly higher mean blood loss in the DFR group (448 ± 211 mL) compared with the locked lateral plating group (319 ± 174 mL; p < 0.01). Tandon T et al. [11] reported DFR blood loss of 400 mL versus 800 mL for ORIF. Conversely, Ross LA et al. [10] reported lower blood loss with DFR (440 mL) compared with LLP (640 mL), although this difference was not statistically significant (p = 0.076).

Infection rates by fixation type varied. Lizcano JD et al. [21] reported eight infectious complications in 54 DFR patients, predominantly systemic infections, including shock/sepsis, compared with only one infection in 45 ORIF patients. Abboud J et al. [15] reported a higher infection rate in the locked plate group (11%) compared with the IMN group (5.8%). Battut T et al. [16] reported infection as part of overall morbidity, with a one-year mortality rate of 12% for implant exchange versus 33% for internal fixation (p < 0.03).

Non-union rates were heavily dependent on fixation type and patient factors. Lizcano JD et al. [21] reported no non-unions in 54 DFR patients versus nine non-unions in 45 ORIF patients (20%). Kriechling P et al. [22], using the term distal femoral arthroplasty (DFA) synonymously with DFR, reported no non-unions in the DFA group versus nine non-unions in the single-plating group. Battut T et al. [16] reported comparable non-union rates between implant exchange (35%) and internal fixation (22%). Aldrian S et al. [14] reported non-unions in seven of 48 patients treated with angular stable plates (14.6%) versus three of 36 patients treated with rigid interlocking nails (8.3%) (Table 5).

**Table 5:** Subgroup analysis by fixation type: plate fixation versus RIMN versus dual plating. RIMN: Retrograde intramedullary nailing; LCP: Locking compression plate; LLP: Lateral locking plate; DFR: Distal femoral replacement; ORIF: Open reduction and internal fixation; mL: millilitres; WB: Weight-bearing.

Fixation Type	Number of Studies	Key Findings
Plate fixation (locking plate, LCP, LLP)	13	Most commonly reported fixation method; mortality varied from 4% to 26.8%; reported blood loss ranged from 319 to 640 mL.
Retrograde intramedullary nailing (RIMN)	12	Generally associated with earlier progression to weight-bearing; reported blood loss ranged from 174 to 483 mL; hospital stay ranged from 6 to 16 days.
Dual plating/other constructs	6	Included double plating and nail-plate combination constructs; mainly used for complex fractures.

Detailed DFR versus ORIF comparison: Periprosthetic joint infection (PJI) was sparsely reported across studies. Ross LA et al. [10] reported one deep infection in the DFR group versus three in the ORIF group. Hoellwarth JS et al. [18] reported one deep infection in the DFR group versus three in the locked plating group. Fu P et al. [19] reported one infection in each group. Darrith B et al. [20] reported one deep infection in the DFR group versus three in the ORIF group. Overall, the PJI data were too limited for meaningful pooled analysis.

Implant failure and aseptic loosening data were inconsistently reported. Lizcano JD et al. [21] reported mechanical complications, including implant failure, as part of their composite outcome. Hoellwarth JS et al. [18] evaluated implant survivorship and reported no statistically significant difference in aseptic loosening between the DFR and locked plating groups. Kriechling P et al. [22] reported revision for failure in 3.3% of DFR patients versus higher rates in both plating groups.

Malunion was reported in four studies. Lizcano JD et al. [21] and Battut T et al. [16] reported comparable malunion rates between groups. Virkus W et al. [23] reported malunion in two of 36 RIMN patients (5.6%) versus two of 12 ORIF patients (16.7%). Gausden EB et al. [24] assessed angular deformity and malalignment as primary outcomes in their plating versus RIMN comparison.

Functional scores were reported in a minority of studies; full data for KSS, OKS, ROM, and KSFS are summarized below.

Return to Daily Activities and Weight Bearing

Postoperative weight-bearing protocols differed substantially between treatment groups. Immediate full weight-bearing was permitted in the DFR group in 10 of 13 studies (76.9%) that reported this outcome. Specifically, Ross LA et al. [10], Hoellwarth JS et al. [18], Leino OK et al. [12], Darrith B et al. [20], Lizcano JD et al. [21] (88.9% weight-bearing as tolerated), Ruder JA et al. [13], Kriechling P et al. [22], Battut T et al. [16] (84.2% of implant exchange patients), and Virkus W et al. [23] all permitted immediate weight-bearing in the DFR or arthroplasty group.

Time to full weight-bearing in the DFR group ranged from 0 days, indicating immediate weight-bearing, to 1.5 days [11]. Kriechling P et al. [22] reported immediate unrestricted postoperative weight-bearing for DFA patients. Leino OK et al. [12] reported immediate full weight-bearing and mobilization for all periprosthetic fracture patients, including those with revision TKA implants, as pain permitted.

In contrast, ORIF groups generally required protected weight-bearing protocols. Darrith B et al. [20] specified protected weight-bearing for 6-12 weeks following ORIF. Tandon T et al. [11] reported a median time to full weight-bearing of 11 weeks in the ORIF group compared with 1.5 days in the DFR group. Abboud J et al. [15] reported a time to full weight-bearing of 7 ± 2 weeks for locked plate fixation and 1 ± 2 weeks for IMN. Battut T et al. [16] reported a median of eight weeks (range, 0-30 weeks) for the internal fixation group versus immediate weight-bearing for the implant exchange group. Aldrian S et al. [14] specified a minimum of 6-8 weeks of protected weight-bearing following internal fixation.

Postoperative rehabilitation protocols were sparsely described. Hoellwarth JS et al. [18] reported that most patients with DFR or locked plating were encouraged to bear full weight immediately. Fu P et al. [19] allowed weight-bearing on postoperative day 2 in the DFR group. Darrith B et al. [20] noted that DFR allowed immediate weight-bearing, while ORIF required protected weight-bearing for 6-12 weeks. Return-to-activity and return-to-work data were not systematically reported across studies. The early weight-bearing advantage of DFR offers an important rehabilitation window, especially for frail elderly patients, in whom prolonged immobilization is associated with significant risks of sarcopenia, thromboembolic events, and functional decline. However, earlier ability to bear weight does not necessarily imply better long-term functional recovery and should not be misconstrued as overall functional superiority of DFR over ORIF (Table 6).

**Table 6:** Return to daily activities and weight-bearing status. Postoperative rehabilitation and mobilization protocols. DFR: Distal femoral replacement; ORIF: Open reduction and internal fixation; WB: Weight-bearing; WBAT: Weight-bearing as tolerated; NWB: Non-weight-bearing; LP: Locking plate; IMN: Intramedullary nail; RIMN: Retrograde intramedullary nail; IE: Implant exchange; IF: Internal fixation; rehab: Rehabilitation; postop: Postoperative; wks: Weeks; NR: Not reported.

Study	Immediate WB in DFR/DFA/IE Group	DFR/DFA/IE Protocol: Time to Full WB and Rehabilitation	ORIF/Fixation Protocol: Time to Full WB and Rehabilitation	Return to Activity
De Marco D et al. (2022) [17]	Yes	Immediate postoperative full WB was allowed directly after surgery.	≥30 days postoperatively; NWB for a minimum of 30-60 days.	DFR: immediate WB; ORIF: NWB for 30-60 days.
Ross LA et al. (2021) [10]	Yes	NR	NR	NR
Hoellwarth JS et al. (2018) [18]	Yes	Immediate WB was encouraged in the DFR group.	Immediate WB was also encouraged in the LLP group.	NR
Tandon T et al. (2020) [11]	Yes	Full WB at 1.5 days.	Full WB at 11 weeks.	NR
Fu P et al. (2022) [19]	Yes	Early WB from postoperative day 2; active knee motion was encouraged; WBAT.	Active knee motion was encouraged; partial WB was started at 4 weeks.	NR
Leino OK et al. (2015) [12]	Yes	Immediate full WB and mobilization, pain permitting.	Immediate mobilization; approximately 6 weeks of light or no WB.	NR
Darrith B et al. (2020) [20]	Yes	Immediate WB was permitted.	Protected WB for 6-12 weeks.	NR
Lizcano JD et al. (2025) [21]	Yes; 88.9% WBAT	NR	NR	NR
Ruder JA et al. (2017) [13]	Yes; WBAT	NR	NR	NR
Kriechling P et al. (2024) [22]	Yes; unrestricted postoperative WB in the DFA group	Immediate unrestricted WB.	Immediate WB was reported for fixation groups.	NR
Abboud J et al. (2024) [15]	Not applicable; no DFR/DFA/IE group	NR	LP: 7 ± 2 weeks with progressive WB rehabilitation; IMN: 1 ± 2 weeks with immediate progressive WB rehabilitation.	NR
Battut T et al. (2022) [16]	Yes; IE: 84.21%	IE: immediate WB, median 0 weeks.	IF: median 8 weeks, range 0-30 weeks.	NR
Virkus W et al. (2022) [23]	Not applicable; no DFR/DFA/IE group	NR	Postoperative WB depended on fixation stability; RIMN: 50% early WBAT; ORIF: 14% early WBAT.	NR
Gausden EB et al. (2021) [24]	Not applicable; no DFR/DFA/IE group	NR	NR	NR
Aldrian S et al. (2013) [14]	Not applicable; no DFR/DFA/IE group	NR	6-8 weeks of protected WB; postoperative rehabilitation was routinely started on days 2-3.	One-year postoperative follow-up reported.

Publication Bias

**Figure 9:**
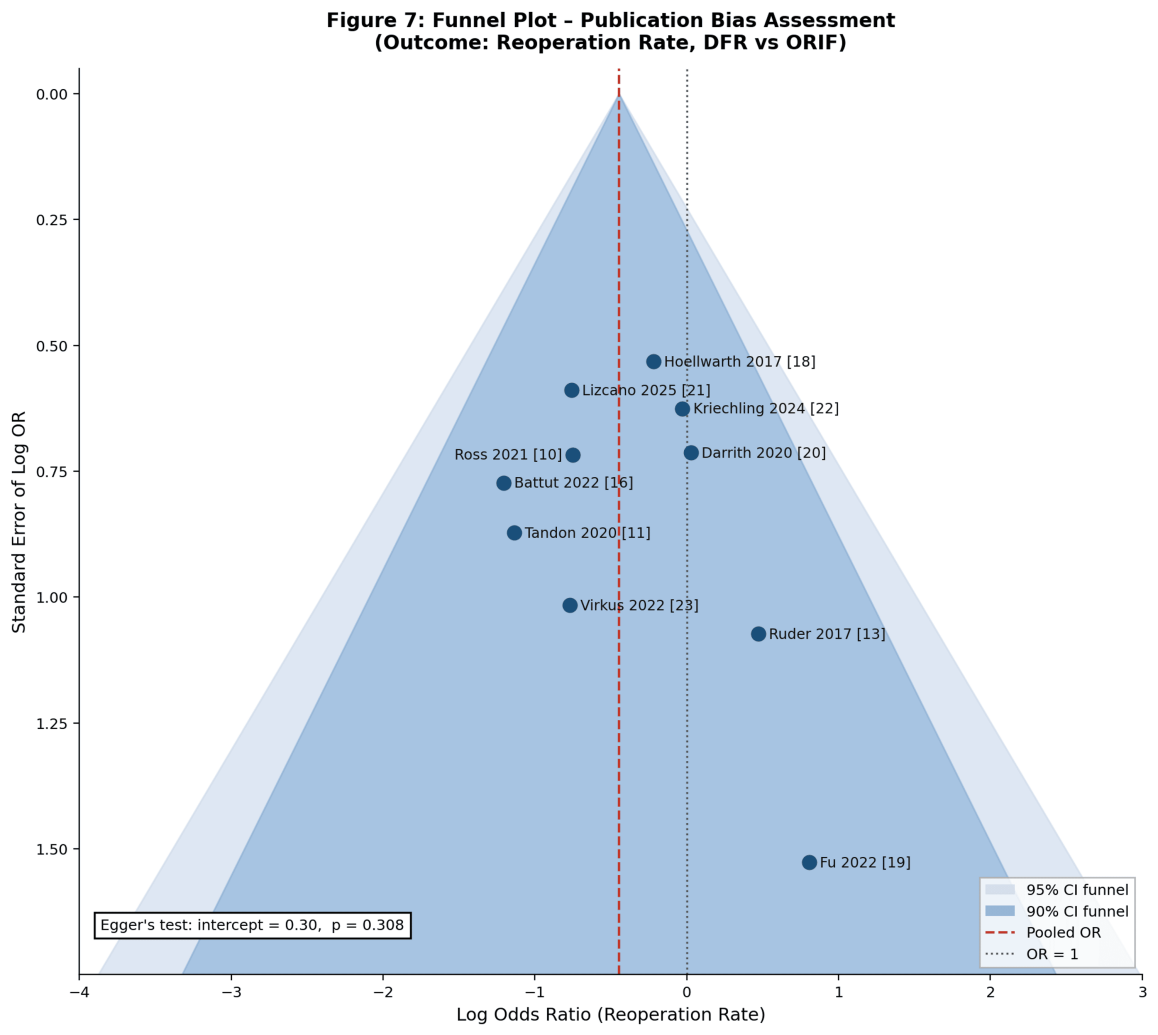
Funnel plot assessing publication bias for the primary outcome, reoperation rate. Egger’s test intercept was 0.30 (p = 0.308). The dashed red line represents the pooled odds ratio, and the dotted line represents OR = 1.

Publication bias was assessed using a funnel plot for the primary outcome of reoperation rate (Figure 9). Visual inspection of the funnel plot revealed a relatively symmetrical distribution of studies around the pooled effect estimate. The Egger regression test for funnel plot asymmetry yielded an intercept of 0.30 (p = 0.308), which was not statistically significant, suggesting no evidence of small-study effects or publication bias for the primary outcome.

All 10 studies fell within the 95% CI funnel boundaries. Studies with smaller standard errors and larger sample sizes, including Hoellwarth JS et al. [18] (n = 140), Kriechling P et al. [22] (n = 111), and Lizcano JD et al. [21] (n = 99), clustered near the pooled effect estimate. Smaller studies, including Fu P et al. [19] (n = 18) and Battut T et al. [16] (n = 52), showed greater dispersion but remained within the funnel boundaries. Overall, the available evidence does not suggest that publication bias substantially affected the pooled estimate for reoperation rate. However, the funnel plot and Egger test have limited power with only ten contributing studies; therefore, publication bias cannot be excluded with certainty.

Additional Clinical Outcomes

Functional outcomes: Functional outcome data were limited and reported heterogeneously. The KSS was reported in three studies. Tandon T et al. [11] reported a mean KSS of 70 in the DFR group versus 68 in the ORIF group at a minimum 72-month follow-up. Fu P et al. [19] reported a KSS of 78.0 ± 2.5 in the DFR group versus 80.3 ± 8.3 in the ORIF group. Darrith B et al. [20] reported identical median KSS scores of 85 in both groups at a mean follow-up of 55-58 months. Brady T et al. [1] additionally reported KSFS, a patient-reported subscore reflecting walking distance, stair-climbing capacity, and the need for walking aids, in two studies, with values significantly higher in the ORIF group than in the DFR group (ORIF: 53 and 52; DFR: 39 and 37; p = 0.012 and p = 0.027, respectively); however, overall KSS was comparable between groups across the same studies.

The OKS was reported in three studies. De Marco D et al. [17] reported an OKS of 33.5 in the DFR group versus 25.9 in the ORIF group at a mean 10.8-month follow-up. Tandon T et al. [11] reported an OKS of 28 in the DFR group versus 27 in the ORIF group. Hoellwarth JS et al. [18] reported an OKS of 35 in the DFR group only.

ROM data were reported in three studies. De Marco D et al. [17] reported a mean ROM of 82.5° (range, 50°-100°) in the DFR group versus 71.1° (range, 50°-90°) in the ORIF group. Tandon T et al. [11] reported ROM of 3.5°-95° in the DFR group versus 5°-85° in the ORIF group. Virkus W et al. [23] reported an equivalent mean ROM of 88° in both the RIMN and ORIF groups. Patient satisfaction scores were infrequently reported and could not be synthesized. Brady T et al. [1] reported that despite earlier weight-bearing permission following DFR, patients treated with DFR were significantly less likely to achieve unassisted ambulation postoperatively compared with those treated with ORIF under the common-effect model (RR 0.60; p = 0.02), although this finding was substantially heterogeneous (I² = 66%) and should be interpreted with caution.

Mortality: Thirty-day mortality was reported in three studies. Ross LA et al. [10] reported 30-day mortality of 6% in the ORIF group versus 0% in the DFR group, with an overall rate of 3.3%. Fu P et al. [19] reported 30-day mortality of 16.7% in the DFR group versus 8.3% in the ORIF group. Lizcano JD et al. [21] reported 30-day mortality of 3.7% in the DFR group versus 4.4% in the ORIF group, with an overall rate of 4.0%.

One-year mortality rates varied substantially across studies, ranging from 4% in Lizcano JD et al. [21] to 33% in Battut T et al. [16] in the internal fixation subgroup; the latter reflects a subgroup with a higher comorbidity burden and is not directly comparable to the pooled cohort. This wide range reflects the heterogeneous frailty and comorbidity profiles of the included patient populations rather than a treatment effect. Hoellwarth JS et al. [18] reported one-year mortality of 22% for locked lateral plating versus 10% for DFR (p = 0.41). Darrith B et al. [20] reported an overall one-year mortality of 26.8%. Leino OK et al. [12] reported an overall one-year mortality of 23.1%. Ruder JA et al. [13] reported an overall one-year mortality of 20.6%. Kriechling P et al. [22] reported an overall one-year mortality of 18%, with rates of 13% in both the DFA and double-plating groups versus 21% in the single-plating group.

Revision and reoperation: Revision and reoperation rates were defined in varying ways across studies. Lizcano JD et al. [21] reported a reoperation rate of 13% in the DFR group (7/54) versus 24.4% in the ORIF group (11/45). Ruder J et al. [13] reported reoperation rates of 8.7% in the DFR group versus 5.7% in the ORIF group. Kriechling P et al. [22] reported revision for refracture in 3.3% of DFA patients. Hoellwarth JS et al. [18] reported reoperation rates of 10% for DFR versus 22% for locked lateral plating (p = 0.41). Leino OK et al. [12] reported a 15.4% reoperation rate for non-union in the DFR group and 15% in the ORIF group.

Discussion

This review synthesized 15 retrospective comparative studies encompassing 1,417 patients to compare DFR with ORIF for the treatment of PDFFs. The principal finding is that DFR confers a statistically significant reduction in the risk of reoperation compared with ORIF (OR 0.64; 95% CI: 0.42-0.97). Secondary outcomes, including mortality, infection, blood loss, hospital stay, and functional scores, did not demonstrate statistically significant or clinically meaningful differences between interventions. Critically, no randomized controlled trial evidence currently exists in this area; collectively, these findings suggest that DFR represents a valuable surgical option in selected patients. However, the evidence base does not support its universal adoption over ORIF. Several trends and methodological considerations warrant careful interpretation.

The statistically significant reduction in reoperation observed with DFR (OR 0.64; 95% CI: 0.42-0.97) represents the most clinically important finding of this review and is consistent with a plausible biological rationale. DFR bypasses the requirement for fracture healing entirely, thereby eliminating the principal drivers of reoperation following ORIF, including non-union, malunion, hardware failure, implant loosening secondary to fixation failure, and fracture re-displacement. In contrast, ORIF, whether performed with lateral locked plates, RIMN, or dual plating constructs, depends on biological union occurring in an often osteoporotic and potentially poorly vascularized fracture bed, a process that may be impaired in frail elderly patients with multiple comorbidities.

This finding is supported by several related studies and reviews cited in this analysis. Brady T et al. [1] reported a pooled reoperation incidence of 12% in ORIF patients versus 7% in DFR patients, a difference that reached statistical significance (p = 0.048), providing independent corroboration from a contemporaneous systematic review. Similarly, Keenan OJ et al. [25] reported that nearly one in five patients undergoing LLP fixation required reoperation (18.6%), with medial comminution and non-anatomic reduction identified as independent predictors of fixation failure, highlighting the vulnerability of ORIF in technically challenging cases. Conversely, Bundschuh KE et al. [8] and Quinzi DA et al. [9] reported comparable reoperation rates between DFR and ORIF in their pooled analyses, underscoring that this advantage is not universally demonstrated and may depend on patient selection and the definition of reoperation.

Several methodological considerations temper this finding. The absolute risk reduction in reoperation with DFR may partly reflect a salvage effect, whereby DFR represents a terminal or near-terminal surgical option with fewer remaining revision possibilities, rather than a true biological advantage. Brady T et al. [1] explicitly raised this concern, noting that surgeons may initially trial fixation and convert to DFR upon failure, thus inflating the ORIF reoperation rate. Additionally, confounding by indication is a major concern across all included studies: patients selected for DFR generally exhibit more severe fracture comminution, poorer bone stock, loose femoral components, and higher comorbidity burdens than those offered ORIF. Darrith B et al. [20] and Lizcano JD et al. [21] both documented significantly higher CCI scores in DFR cohorts, reflecting a systematic tendency to select DFR for higher-risk patients who may paradoxically be more likely to experience complications. That the reoperation advantage for DFR persisted despite these baseline disadvantages is noteworthy, although this does not rule out residual confounding.

One-year mortality showed a trend favoring DFR (OR 0.70; 95% CI: 0.45-1.09) that did not reach statistical significance, with negligible heterogeneity across studies (I² = 0.0%). This pattern should be interpreted with considerable caution. The non-significant trend may reflect a modest, true mortality benefit conferred by avoiding prolonged immobilization in frail elderly patients, given the established relationship between early mobilization and reduced mortality after lower-limb fragility fractures. However, it may equally represent a statistical artifact of inadequate power, as mortality events were relatively infrequent in most individual studies.

Mortality in this patient population is predominantly driven by pre-existing frailty, comorbidity burden, baseline ambulatory status, and the physiological consequences of major surgery in elderly patients, rather than by the specific implant used. Bundschuh KE et al. [8] observed significantly higher medical complication rates following DFR (23.1% versus 8.5% for ORIF, p = 0.0006), suggesting that the greater surgical insult of prosthetic replacement, combined with the typically frailer patient cohort selected for DFR, may offset any mobility-related survival benefit. Similarly, Quinzi DA et al. [9] found no statistically significant difference in one-year mortality among the three treatment modalities, ORIF, IMN, and DFR, with rates of approximately 5%-6% across groups in the subset of studies reporting this outcome. These findings collectively suggest that mortality outcomes in this population are multifactorial and unlikely to be decisively influenced by the choice between DFR and ORIF alone. Claiming a mortality benefit for DFR based on currently available evidence is not justified.

The pooled analysis of infection rates yielded an OR of 1.39 (95% CI: 0.61-3.18), indicating a non-significant trend toward higher infection risk with DFR. Although not statistically significant, this trend is biologically plausible and clinically important. DFR requires more extensive surgical exposure than most ORIF approaches, involves prosthetic reconstruction with foreign material of greater surface area, and is associated with longer operative times in many series. These factors combine to increase the theoretical risk of PJI, which, in the context of a megaprosthesis, carries particularly severe consequences, including the need for multi-stage revision, arthrodesis, or even amputation in refractory cases.

This concern is echoed in the comparative literature. Quinzi DA et al. [9] identified a statistically significantly higher rate of deep infection in DFR patients relative to both ORIF and IMN groups (9% versus 5%-6%, p = 0.03) in their pooled systematic review of 52 studies, representing one of the largest analyses of this outcome in the literature. The observation by Bundschuh KE et al. [8] that deep infection can progress to chronically infected non-unions or require prosthetic salvage in a substantial proportion of cases underscores the qualitatively different, and often more morbid, consequences of infection in the DFR setting compared with ORIF. Brady T et al. [1] reported a non-significant infection risk ratio of 1.10 (95% CI: 0.60-2.01), consistent with the present findings.

This observed infection risk should, however, be balanced against the mechanical complication profile of ORIF. Failure of internal fixation, encompassing non-union, implant breakage, screw pullout, and progressive malunion, represents a qualitatively different but equally serious complication category that may also require reoperation with considerable morbidity. The decision between DFR and ORIF, therefore, involves a clinical trade-off between the risk of infection, which is more inherent to prosthetic reconstruction, and the risk of mechanical failure, which is more inherent to fracture fixation. This balance is best navigated through careful patient and fracture selection rather than a universal preference for either intervention.

The pooled OR for non-union comparing DFR with ORIF was 0.86 (95% CI: 0.37-1.99), which was not statistically significant, with moderate heterogeneity (I² = 53.8%). This result must be interpreted with particular caution, as non-union is a biologically and conceptually irrelevant outcome for DFR: prosthetic reconstruction, by definition, does not require fracture healing. It therefore cannot result in non-union in the traditional sense. The inclusion of studies reporting zero non-unions in DFR groups alongside studies reporting rates of 20%-35% in ORIF groups directly drives the observed heterogeneity and renders a direct pooled comparison of limited biological meaning.

The more clinically relevant comparison is between non-union following ORIF and implant-related failure following DFR. For ORIF, non-union remains a substantive risk, particularly in the context of severe comminution, distal fractures with limited fixation surface, osteoporotic bone, and suboptimal surgical technique. Al-Jabri T et al. [5] reported an overall non-union rate of 9.4% across 519 patients undergoing locking compression plating or RIMN for PDFFs, with no significant difference between the two fixation methods. Keenan OJ et al. [25] specifically identified medial comminution as an independent predictor of non-union and reoperation following LLP fixation (hazard ratio 10.7, p = 0.020). At the same time, anatomic reduction was independently protective (hazard ratio 0.11, p = 0.046). These findings emphasize that non-union following ORIF is not an inevitable outcome but is critically dependent on fracture characteristics and surgical execution.

Modern fixation strategies, including dual plating and nail-plate combination techniques, may further mitigate the risk of non-union in ORIF. Bundschuh KE et al. [8] noted that dual plating for PDFFs has been associated with union rates approaching 100% in selected small series and that augmented fixation constructs may facilitate early weight-bearing even following ORIF. These advances in fixation technique reduce, but do not eliminate, the biological advantages of DFR in cases with severe comminution or extremely distal fracture patterns.

The pooled MD in intraoperative blood loss was -56.2 mL (95% CI: -115.4 to 2.9 mL), favoring DFR but not reaching statistical significance. Very high heterogeneity (I² = 90.4%) renders this estimate unreliable as a guide to clinical expectations. The direction of individual study effects was inconsistent: Tandon T et al. [11] reported markedly lower blood loss with DFR (MD -400 mL), while Hoellwarth JS et al. [18] reported significantly higher blood loss with DFR (MD +129 mL). Brady T et al. [1] reported descriptive weighted means suggesting lower blood loss with DFR (411.7 mL versus 601.3 mL for ORIF). However, they were unable to perform a formal meta-analysis due to the lack of variance data in most of the included studies. This inconsistency likely reflects genuine differences in surgical approach, the extent of soft-tissue mobilization for each procedure, individual surgeons’ techniques, intraoperative cell-salvage use, and variable transfusion practices across institutions. Blood loss should not be used as a primary driver of treatment selection in clinical practice.

Length of hospital stay showed a similarly negligible pooled difference (MD -0.1 days; 95% CI: -1.0 to 0.8 days) with very high heterogeneity (I² = 82.4%), confirming the absence of a clinically meaningful difference between procedures when measured in aggregate. Brady T et al. [1] likewise reported no statistically significant pooled difference in length of stay (MD 0.53 days; 95% CI: -1.53 to 2.59; I² = 54.4%). These outcomes are profoundly influenced by institutional discharge criteria, the structure of the healthcare system, the availability of rehabilitation facilities, and patient frailty. These factors vary substantially across the seven countries represented in the included studies. Interpreting these outcomes in the context of a single institution or healthcare system, based on pooled international data, would be inappropriate.

One of the most clinically relevant distinctions between DFR and ORIF in this patient population concerns the timing of postoperative weight-bearing. Across the included studies, immediate or very early full weight-bearing was permitted in the DFR group in 76.9% of studies reporting this outcome, compared with protected weight-bearing protocols, often ranging from 6 to 12 weeks, in ORIF groups. Brady T et al. [1] reported a significantly shorter weighted mean time to weight-bearing with DFR (2 days) compared with ORIF (78 days; p = 0.04), representing perhaps the most tangible perioperative advantage of prosthetic reconstruction. The clinical importance of this difference is potentially substantial: prolonged immobilization in frail elderly patients with periprosthetic fractures is associated with sarcopenia, thromboembolic events, pressure injuries, pneumonia, delirium, and loss of pre-injury functional independence.

However, two important qualifications temper this advantage. First, Keenan OJ et al. [25] demonstrated in a retrospective comparative cohort that immediate unrestricted weight-bearing as tolerated following LLP fixation of PDFFs was not associated with an increased risk of fixation failure or reoperation compared with six weeks of restricted weight-bearing (relative risk 1.03; p = 0.91). Wardle B et al. [7] similarly found no statistically significant difference in complication rates, including non-union, malunion, infection, and mortality, between early and delayed weight-bearing groups across 844 patients with geriatric distal femoral fractures managed by fixation. These findings suggest that modern fixation constructs, particularly when augmented with dual plating or nail-plate combinations and with adequate reduction, may safely facilitate early weight-bearing in selected patients, narrowing the distinction from DFR in this domain.

Second, Brady T et al. [1] observed that despite earlier weight-bearing permission after DFR, patients treated with DFR were significantly less likely to achieve unassisted ambulation postoperatively compared with those treated with ORIF under the common-effect model (RR 0.60; p = 0.02), although this finding was substantially heterogeneous (I² = 66%) and should be interpreted with caution. One included study noted that despite immediate full weight-bearing being permitted, almost none of the DFR patients were able to do so at discharge [1]. This observation suggests that permission for immediate weight-bearing does not automatically translate into its achievement, particularly in the frailest patients, and that the anticipated mobilization advantage of DFR may be partially illusory in practice.

Functional outcomes were sparsely and inconsistently reported across the included studies, with KSS, OKS, and ROM data available in only three studies each. No pooled analysis was possible. The available point estimates did not consistently favor either treatment: KSS values were similar across groups in the three reporting studies; OKS was slightly higher in the DFR group in one study but nearly identical in two others; and ROM was reported variably. This is consistent with the findings of Brady T et al. [1], who reported no significant difference in OKS between DFR and ORIF (MD = -0.11; p = 0.85) across four studies, with minimal heterogeneity. Al-Jabri T et al. [5] similarly noted that PROM data across their included studies were too heterogeneous, employing nine different scoring instruments across six studies, to allow meaningful synthesis.

Of particular interest is the observation by Brady T et al. [1] that KSFS, a patient-reported subscore reflecting self-assessed functional capacity, including walking distance, stair climbing, and need for walking aids, was significantly higher in ORIF patients in the two studies reporting this metric (ORIF: 53 and 52; DFR: 39 and 37; p = 0.012 and p = 0.027, respectively), despite overall KSS being comparable. This finding, if replicated, would suggest that the lived functional experience of patients following ORIF may exceed that following DFR, perhaps reflecting the greater surgical insult and soft-tissue disruption associated with prosthetic reconstruction, or that DFR is disproportionately applied to patients with pre-existing functional limitations. However, the very limited data preclude firm conclusions, and these findings must be interpreted within the context of potential selection bias and small sample sizes. Current evidence does not support a claim of long-term functional superiority for either DFR or ORIF, and the early weight-bearing advantage of DFR does not appear to translate reliably into superior long-term function.

The findings of this review support a nuanced, patient-centered approach to surgical decision-making in PDFFs. DFR may be the preferred strategy in patients presenting with severe distal comminution or fractures that preclude stable internal fixation, a loose or failed femoral component necessitating revision arthroplasty, markedly poor bone stock or osteoporotic bone incompatible with reliable fixation, pre-existing significant functional limitations or low ambulatory status, or an inability to comply with protected weight-bearing protocols due to cognitive impairment, neurological conditions, or extreme frailty. In these settings, the avoidance of fracture-dependent healing and the facilitation of early mobilization represent meaningful clinical advantages that may outweigh the higher infection risk and greater surgical morbidity of prosthetic reconstruction.

ORIF remains a reasonable and appropriate strategy in patients with fractures amenable to stable reconstruction, particularly those involving Su type I and II fracture patterns, a well-fixed femoral component, adequate distal bone stock for secure screw purchase, and a clinical trajectory compatible with protected weight-bearing rehabilitation. Modern ORIF techniques, including dual plating, nail-plate combinations, and augmented locking plate constructs, have extended the applicability of fixation to more distal and complex fracture patterns while potentially facilitating earlier weight-bearing than traditional protocols. Bundschuh KE et al. [8] noted that advances in fixation technique may continue to expand the utility of ORIF, and the decision to proceed with DFR should not be made reflexively based solely on fracture complexity.

Treatment selection should be individualized based on a comprehensive assessment of fracture pattern and classification, femoral component stability and design, bone quality and quantity, patient age, frailty, comorbidity burden, pre-injury functional status and ambulatory capacity, ability to comply with rehabilitation, available surgical expertise, and institutional resources. A multidisciplinary approach involving orthopedic trauma surgeons, arthroplasty specialists, anesthesiologists, and geriatricians is likely to optimize perioperative management in this vulnerable patient population, consistent with the care standards established for hip fractures in elderly patients.

Strengths and limitations

This systematic review and meta-analysis has several methodological strengths. The review encompasses the largest combined patient cohort to date comparing DFR and ORIF for PDFFs (n = 1,417), providing greater statistical power in the primary outcome analysis than previous single-center or smaller pooled studies. Multiple clinically relevant outcomes were examined, providing a comprehensive comparative picture across domains of safety, efficacy, and function. The use of a random-effects model, assessment of heterogeneity using I² statistics, subgroup analysis by fixation type, and formal publication bias assessment through funnel plots and Egger's test represent methodological rigor appropriate to the available evidence. The identification of negligible heterogeneity in the primary reoperation analysis (I² = 0%) strengthens confidence in the pooled estimate.

However, important limitations must be acknowledged. All 15 included studies were retrospective in design, and none reported prospective data collection or randomization, representing the lowest tier of comparative evidence. MINORS scores ranged from 12 to 20/24, reflecting fair-to-good methodological quality, but common weaknesses across studies, including lack of unbiased endpoint assessment, loss to follow-up exceeding 5%, and absence of prospective sample size calculations, limit confidence in individual study results. The most consequential limitation is confounding by indication: patients selected for DFR consistently exhibited greater fracture complexity, worse bone stock, higher comorbidity burden, and poorer pre-injury function than those offered ORIF. Despite the pooled benefit of DFR in reoperation rates persisting across studies, residual confounding from unmeasured variables cannot be excluded in the absence of randomized or propensity-matched data.

Additional limitations include marked heterogeneity in fracture classification systems across studies, variation in ORIF technique, including pooling of locked plate, RIMN, and dual plating within a single ORIF comparator group in most studies, inconsistent rehabilitation protocols and weight-bearing prescriptions, heterogeneous definitions of reoperation and complications across studies, very limited and inconsistently reported functional outcome data, and the inability to perform patient-level subgroup analyses by fracture pattern, bone quality, or implant characteristics. Follow-up duration also varied widely, from a minimum of six weeks to six years, precluding assessment of longer-term implant survivorship, component loosening, or late functional decline.

Future research

The findings of this review identify several priorities for future research. Prospective, multicenter studies with pre-specified protocols and standardized outcome definitions are urgently needed. A well-powered multicenter randomized controlled trial or prospective registry study comparing DFR and ORIF in clearly defined patient and fracture subgroups would substantially advance the evidence base beyond the currently available retrospective literature. Such studies should incorporate frailty assessment instruments, comorbidity indices, and pre-injury ambulatory status as covariates to appropriately adjust for confounding by indication.

Standardization of outcome definitions across studies is essential, particularly for reoperation, revision, infection, non-union, implant failure, and functional recovery. The use of patient-reported outcome measures, including OKS, KSFS, EuroQol Five-Dimension questionnaire (EQ-5D), and patient satisfaction instruments, should be mandated as primary or co-primary endpoints in future comparative studies, given the evidence that clinician-assessed and patient-reported functional outcomes may diverge. Longer follow-up periods of at least two to five years are necessary to capture late implant-related events, including aseptic loosening, periprosthetic fractures around DFR components, and late non-union in ORIF patients. Cost-effectiveness analyses comparing the two strategies across different healthcare systems would also provide important decision-support information for health policy and resource allocation.

## Conclusions

This systematic review and meta-analysis demonstrates that DFR is associated with a statistically significant reduction in reoperation compared with ORIF for PDFFs. However, these results should be interpreted as an association rather than proof of superiority, given the retrospective evidence base, confounding by indication, and the possibility that lower reoperation rates partly reflect the salvage nature of prosthetic reconstruction. These findings support the selective and judicious application of DFR rather than its routine adoption. Treatment selection must remain individualized, with ORIF appropriate for reconstructible fractures with stable femoral components and adequate bone quality, and DFR reserved for patients in whom reliable fixation is unlikely or compliance with protected weight-bearing is unrealistic.

One-year mortality showed a non-significant trend favoring DFR, while infection showed a non-significant trend toward higher rates in the DFR group. Neither blood loss, hospital stay, nor overall functional scores demonstrated clinically meaningful or statistically significant differences between the two treatments, although the KSFS, a patient-reported subscore of walking capacity and aid use, was significantly higher in ORIF patients in two studies reporting this metric, a finding that warrants replication. DFR appears to confer a meaningful advantage in early weight-bearing rehabilitation, although this does not reliably translate into superior long-term ambulatory outcomes.

DFR should be considered a valuable surgical option in carefully selected high-risk patients, including those with severe comminution, loose femoral components, poor bone stock, or inability to comply with protected weight-bearing. ORIF, including modern augmented fixation constructs, remains appropriate in patients with reconstructible fractures, stable implants, adequate bone quality, and the capacity to follow structured rehabilitation. Individualized surgical decision-making, guided by fracture characteristics, patient frailty, and institutional expertise, remains paramount. Stronger prospective evidence, including randomized controlled trials and standardized registry studies, is needed to definitively establish the optimal treatment strategy for this challenging injury.

## Appendices

Appendix 1

**Table 7:** Subgroup analysis: DFR versus ORIF comparison. Clinical outcomes, complications, and functional scores. DFR: Distal femoral replacement; ORIF: Open reduction and internal fixation; LLP: Lateral locking plate; LP: Locking plate; IMN: Intramedullary nail; RIMN: Retrograde intramedullary nail; IE: Implant exchange; IF: Internal fixation; DP: Double plating; SP: Single plating; ASP: Angular stable plate; RIN: Rigid interlocking nail; PJI: Periprosthetic joint infection; KSS: Knee Society Score; OKS: Oxford Knee Score; NR: Not reported.

Study	N (Groups Compared)	Mortality (30-day vs. 1-year)	Infection	PJI	Implant Failure	Non-union	Malunion	Functional Scores
De Marco D et al. (2022) [17]	DFR: 4; ORIF: 9	NR	NR	NR	DFR: 5; ORIF: NR	NR	NR	OKS: 33.5 vs. 25.9
Ross LA et al. (2021) [10]	DFR: 27; LLP: 33	30-day: DFR 0%, LLP 6%; 1-year: DFR 11%, LLP 15%	DFR: 1; LLP: 3	NR	NR	DFR: 0; LLP: 5	NR	NR
Hoellwarth JS et al. (2018) [18]	DFR: 53; LLP: 87	30-day: NR; 1-year: DFR 10%, LLP 22%	DFR: 1; LLP: 3	DFR: 1; LLP: 3	NR	NR	NR	OKS: DFR 35
Tandon T et al. (2020) [11]	DFR: 21; ORIF: 40	NR	DFR: 1; ORIF: 2	DFR: 0; ORIF: 1	DFR: 0; ORIF: 1	DFR: 0; ORIF: 1	DFR: 0; ORIF: 6	KSS: 70 vs. 68; OKS: 28 vs. 27
Fu P et al. (2022) [19]	DFR: 6; LCP: 12	30-day: NR; 1-year: DFR 17%, LCP 8.3%	DFR: 1; LCP: 2	NR	DFR: 0; LCP: 1	NR	NR	KSS: 78.0 ± 2.5 vs. 80.3 ± 8.3
Leino OK et al. (2015) [12]	DFR: 39; ORIF: 29	30-day: NR; 1-year: 23.1%	DFR: 3; ORIF: NR	DFR: 3; ORIF: NR	NR	DFR: 6; ORIF: NR	NR	NR
Darrith B et al. (2020) [20]	DFR: 22; ORIF: 50	30-day: NR; 1-year: 26.8%	DFR: 1; ORIF: 3	DFR: 1; ORIF: 3	NR	NR	NR	KSS: 85 vs. 85
Lizcano JD et al. (2025) [21]	DFR: 54; ORIF: 45	30-day: DFR 3.7%, ORIF 4.4%; 1-year: NR	DFR: 8; ORIF: 1	DFR: 3; ORIF: 1	DFR: 4; ORIF: 10	DFR: 0; ORIF: 9	NR	NR
Ruder JA et al. (2017) [13]	DFR: 23; ORIF: 35	30-day: 5.1%; 1-year: 20.6%	DFR: 1; ORIF: 0	DFR: 0; ORIF: 0	DFR: 0; ORIF: NR	DFR: NR; ORIF: 1	DFR: NR; ORIF: 1	NR
Kriechling P et al. (2024) [22]	DFA: 30; DP: 30; SP: 51	30-day: DFA 0%, DP 6.7%, SP 3%; 1-year: DFA 13%, DP 13%, SP 21%	DFA: 1; plating groups: 2	NR	DFA: 2; DP: 0; SP: 9	DFA: 0; DP: 0; SP: 9	NR	NR
Abboud J et al. (2024) [15]	LP: 365; IMN: 69	30-day: NR; 1-year: LP 22.5%, IMN 13%	LP: 11%; IMN: 5.8%	NR	LP: 6.8%; IMN: 5.7%	NR	NR	NR
Battut T et al. (2022) [16]	IE: 20; IF: 32	30-day: NR; 1-year: IE 12%, IF 33%	NR	NR	NR	IE: 7; IF: 7	IE: 6; IF: 6	NR
Virkus W et al. (2022) [23]	RIMN: 36; ORIF: 12	NR	RIMN: NR; ORIF: 1	NR	RIMN: NR; ORIF: 2	RIMN: 2; ORIF: 2	RIMN: 2; ORIF: 1	NR
Gausden EB et al. (2021) [24]	Plating: 74; RIMN: 23	Mortality: 3 events; timing not specified	UTIs: 25	NR	NR	NR	NR	NR
Aldrian S et al. (2013) [14]	ASP: 48; RIN: 38	NR	ASP: 1 superficial wound infection; RIN: 2 pneumonias	NR	NR	ASP: 3 plate/hardware failures; RIN: 1 distal interlocking screw loosening	ASP: 7; RIN: 3	Functional score: ASP 2.22; RIN 2.20

## References

[1] Brady T, Shapiro S, Barger K, Dehghan N (2026). Open reduction and internal fixation versus distal femoral replacement for periprosthetic distal femur fractures: a systematic review and meta-analysis. OTA Int.

[2] Wadhwa H, Salazar BP, Goodnough LH (2022). Distal femur replacement versus open reduction and internal fixation for treatment of periprosthetic distal femur fractures: a systematic review and meta-analysis. J Orthop Trauma.

[3] Elnewishy A, Elgamal M, Elkohail A (2025). Distal femoral replacement versus open reduction and internal fixation in elderly distal femur fractures: a systematic review and meta-analysis. Cureus.

[4] Rinehart D, Youngman T, Ahn J, Huo M (2021). Review of patient-reported outcomes in periprosthetic distal femur fractures after total knee arthroplasty: a plate or intramedullary nail?. Arthroplasty.

[5] Al-Jabri T, Wood MJ, Faddul F (2024). Periprosthetic distal femoral fractures around a total knee arthroplasty: a meta-analysis comparing locking compression plating and retrograde intramedullary nailing. Orthop Rev (Pavia).

[6] Puga TB, Anderson LF, Olfson ER (2025). Cemented and uncemented (press-fit) fixation in distal femur replacement (DFR): a systematic review and meta-analysis. Advanced Orthopaedics.

[7] Wardle B, Lynch JT, Staniforth T, Ward T, Smith P (2024). Weightbearing versus non-weight bearing in geriatric distal femoral fractures: a systematic review and meta-analysis. Eur J Trauma Emerg Surg.

[8] Bundschuh KE, Grommersch BM, Tipton SC, Chihab S, Wilson JM, Guild GN 3rd (2023). Distal femoral replacement versus operative fixation for periprosthetic distal femur fractures: a systematic review and meta-analysis. J Arthroplasty.

[9] Quinzi DA, Ramirez G, Kaplan NB, Myers TG, Thirukumaran CP, Ricciardi BF (2021). Early complications and reoperation rates are similar amongst open reduction internal fixation, intramedullary nail, and distal femoral replacement for periprosthetic distal femur fractures: a systematic review and meta-analysis. Arch Orthop Trauma Surg.

[10] Ross LA, Keenan OJ, Magill M (2021). Management of low periprosthetic distal femoral fractures. Bone Joint J.

[11] Tandon T, Tadros BJ, Avasthi A, Hill R, Rao M (2020). Management of periprosthetic distal femur fractures using distal femoral arthroplasty and fixation - comparative study of outcomes and costs. J Clin Orthop Trauma.

[12] Leino OK, Lempainen L, Virolainen P, Sarimo J, Pölönen T, Mäkelä KT (2015). Operative results of periprosthetic fractures of the distal femur in a single academic unit. Scand J Surg.

[13] Ruder JA, Hart GP, Kneisl JS, Springer BD, Karunakar MA (2017). Predictors of functional recovery following periprosthetic distal femur fractures. J Arthroplasty.

[14] Aldrian S, Schuster R, Haas N (2013). Fixation of supracondylar femoral fractures following total knee arthroplasty: is there any difference comparing angular stable plate fixation versus rigid interlocking nail fixation?. Arch Orthop Trauma Surg.

[15] Abboud J, Moussa MK, Sader Z, Favreau H, Bégué T, Flecher X, Ehlinger M (2024). Management of periprosthetic femoral fractures following total knee arthroplasties using locking plates or intramedullary nailing. Comparative study of 567 cases. Orthop Traumatol Surg Res.

[16] Battut T, Argenson JN, Flecher X, Le Baron M (2022). Comparison of morbidity-mortality and functional results between implant exchange and internal fixation by plate for periprosthetic femoral fracture in total knee arthroplasty: a 52-case series. Orthop Traumatol Surg Res.

[17] De Marco D, Messina F, Meschini C (2022). Periprosthetic knee fractures in an elderly population: open reduction and internal fixation vs distal femur megaprostheses. Orthop Rev (Pavia).

[18] Hoellwarth JS, Fourman MS, Crossett L, Goodman M, Siska P, Moloney GB, Tarkin IS (2018). Equivalent mortality and complication rates following periprosthetic distal femur fractures managed with either lateral locked plating or a distal femoral replacement. Injury.

[19] Fu P, Liang W, Gao Z, Zheng S, Fan W (2022). Comparison of locking compression plate and distal femoral replacement for periprosthetic distal femoral fractures: a retrospective study. J Int Med Res.

[20] Darrith B, Bohl DD, Karadsheh MS, Sporer SM, Berger RA, Levine BR (2020). Periprosthetic fractures of the distal femur: Is open reduction and internal fixation or distal femoral replacement superior?. J Arthroplasty.

[21] Lizcano JD, Giakas AM, Goh GS, Abbaszadeh A, Reddy YC, Courtney PM (2025). Fix or replace? Comparable outcomes with internal fixation and distal femoral replacement for periprosthetic fractures above total knee arthroplasty. J Arthroplasty.

[22] Kriechling P, Bowley AL, Ross LA, Moran M, Scott CE (2024). Double plating is a suitable option for periprosthetic distal femur fracture compared to single plate fixation and distal femoral arthroplasty. Bone Jt Open.

[23] Virkus W, Lieder C, Jang Y, Rea P, Gaski G (2022). Results of low distal femur periprosthetic fractures. J Orthop Trauma.

[24] Gausden EB, Lim PK, Rabonivich A (2021). Outcomes of periprosthetic distal femur fractures following total knee arthroplasty: intramedullary nailing versus plating. Injury.

[25] Keenan OJ, Ross LA, Magill M, Moran M, Scott CE (2021). Immediate weight-bearing is safe following lateral locked plate fixation of periprosthetic distal femoral fractures. Knee Surg Relat Res.

